# Diet, lifestyle and telomere length: using Copula Graphical Models on NHANES data

**DOI:** 10.18632/aging.206194

**Published:** 2025-01-29

**Authors:** Angelo M. Tedaldi, Pariya Behrouzi, Pol Grootswagers

**Affiliations:** 1 Division of Human Nutrition and Health, Wageningen University and Research, Wageningen, Netherlands; 2 Biometris, Mathematical and Statistical Methods, Wageningen University and Research, Wageningen, Netherlands

**Keywords:** telomere length, NHANES, C-reactive protein, γ-tocopherol, caffeine

## Abstract

Telomere length has been related to human health and ageing in multiple studies. However, these studies have analyzed a small set of variables, according to pre-formulated hypotheses. We used data from NHANES 1999-2002 to perform a preregistered cross-sectional analysis. From these four years we selected the participants with available leukocyte telomere length measure and with plausible daily energy intake, leading to a total study population of 7096 participants. Then, we divided the participants in three groups according to age: Young 20-39 (*n* = 2623), Middle 40-59 (*n* = 2210), Old 60-84 (*n* = 2263). On each group we performed Copula Graphical Modelling (CGM) to capture the links between the variables of interest, and we conducted certainty and sensitivity analyses to understand the robustness of the results. Blood levels of C-reactive protein and γ-tocopherol, and intake of caffeine and fibers are inversely related to telomere length across the age strata. Sex, race, smoking, physical activity and indicators of socioeconomic status have almost no direct connection with telomeres; however, they are directly linked to C-reactive protein, which in turn is connected to leukocyte telomere length. C-reactive protein is therefore a possible central mediator of the effect of these factors on telomeres.

## INTRODUCTION

Telomeres are non-coding DNA sequences repeated in tandem at the end of each chromosome, and they act as a protective cap of coding DNA [1, 2]. At birth, every human being has telomeres that are on average 11kbp long, ranging from a minimum of 8kbp for some individuals to a maximum of 13kbp for others [3]. In the first 20 years of life telomere length decreases at a high rate, and from early adulthood onwards there is a shortening of around 25bp per year in every replicating tissue [4–6].

Once telomeres become short, they are associated with all-cause mortality risk [7], and they are a risk factor of age-related pathologies such as pulmonary fibrosis and bone marrow failure due to anaplastic anemia [8–11]. Also, short telomeres can be inherited as in the disease dyskeratosis congenita [12, 13]. In this case, they are connected to high risk of developing pulmonary fibrosis and bone marrow failure, and they lead to premature ageing and death [14]. It is therefore important to counteract telomere shortening to avoid the onset of these pathologies and of age-related phenotypes.

Despite the fact that we were all born with telomeres of different sizes due to the heritability of telomere length [4, 15, 16], being born with long telomeres is no guarantee of maintaining long telomeres throughout one’s life, because during adulthood the rate of telomere shortening differs among individuals [6, 17]. The differences in telomere length (TL) decline have been linked to dietary and lifestyle factors by many cross-sectional studies [18–20]. Among the lifestyle factors investigated are physical activity, which has a positive effect on TL [21, 22]; and cigarette smoking, BMI and obesity which all have a negative impact on telomeres [19, 23, 24]. As for dietary factors, some of the diets and dietary indices positively related to increased TL are the following: Mediterranean diet [25, 26], plant-rich diets [27], high oxidative score [28], low dietary inflammatory index [29, 30] and high diet quality [20].

However, four main limitations are common to the cross-sectional research that has been conducted over TL and lifestyle: first, only certain variables are selected, and potential mediators, covariates and confounders are excluded; this is evident when physical activity or socioeconomic status are considered while diet is not taken into account [18, 22]. Then, participants with one or more missing variables of interest are not included in the analyses; such exclusion leads to exploiting reduced datasets, up to less than half of the original study sample [28]. Also, leukocyte composition is generally omitted, despite the fact that telomere length is measured in leukocytes. This last point is very important because leukocytes are a group of diverse cell populations that are all characterized by different telomere length, and the composition of this group varies among individuals [6, 31, 32]. Finally, previous studies focus mainly on whole foods and dietary pathway, which are indeed very important, since we eat foods and not nutrients. However, it could be interesting to discover the specific nutrients and food components that are related to TL, because, in this way, new possible biological mechanisms of interactions between diet and TL could be hypothesized.

A statistical tool that can face these three problems at once is Copula Graphical Modelling (CGM). Not only can CGM deal with missing values and thus enable the inclusion of a larger group of participants compared to previous studies; but it can also include a vast number of categorical and continuous variables in the analysis [33, 34]. Thanks to the latter characteristic, it is possible to analyze the impact of diet and lifestyle on telomere length, together with many covariates, mediators and confounders, including leukocyte composition. Moreover, CGM is data-driven and not hypothesis-driven. The main advantage of a data-driven approach is that it permits to explore the relationships among the variables in a dataset, while minimizing the biases related to choices made *a priori*, such as the choice of the variables to be included or to excluded in the model.

At the time of writing and to the best of our knowledge, no study has investigated the relationship between diet, lifestyle and TL using a data-driven approach. In the present work, we apply CGM on the data collected by NHANES in the years 1999-2002. Our aim is to search for relations between TL, food components and other lifestyle factors in a preregistered data-driven way.

## RESULTS

### Display of the results

The first results we are going to show are the general characteristics of the study population. All these characteristics are presented in tables of five columns, where the participants are stratified in quartiles of TL. In Table 1 we give an overview of demographic, lifestyle and examination variables of the entire study population (*n* = 7096), while in the three Supplementary Table 1 we do the same for each of the three age groups: Young (*n* = 2623), Middle (*n* = 2210), Old (*n* = 2263). Following this stratification, in Table 2 and in the Supplementary Table 2 we present the laboratory variables of the entire study population and of the Young, Middle and Old group.

**Table 1:** Demographic, examination and lifestyle characteristics of participants, stratified by telomere length quartiles.^1^

	Q1	Q2	Q3	Q4
*n*	1774	1774	1774	1774
***Telomere length (T/S ratio)***	0.74 (0.08)	0.93 (0.04)	1.08 (0.05)	1.36 (0.16)
***NHANES cycle (%)***				
*1999-2000*	968 (54.6)	794 (44.8)	751 (42.3)	735 (41.4)
*2001-2002*	806 (45.4)	980 (55.2)	1023 (57.7)	1039 (58.6)
***Sex (%)***				
*Female*	821 (46.3)	894 (50.4)	997 (56.2)	986 (55.6)
*Male*	953 (53.7)	880 (49.6)	777 (43.8)	788 (44.4)
***Age (years)***	59 (17)	51 (17)	45 (17)	39 (15)
***Race (%)***				
*Mexican American*	456 (25.7)	449 (25.3)	467 (26.3)	356 (20.1)
*Other Hispanic*	76 (4.3)	85 (4.8)	96 (5.4)	121 (6.8)
*White*	975 (55.0)	909 (51.2)	882 (49.7)	831 (46.8)
*Black*	227 (12.8)	273 (15.4)	270 (15.2)	407 (22.9)
*Other*	40 (2.3)	58 (3.3)	59 (3.3)	59 (3.3)
***Education level (%)***				
*Less Than 9th Grade*	372 (21.0)	276 (15.6)	239 (13.5)	178 (10.0)
*9-11th Grade*	326 (18.4)	302 (17.1)	307 (17.3)	304 (17.2)
*High School Graduate*	404 (22.8)	402 (22.7)	399 (22.5)	450 (25.4)
*Some College or AA degree*	396 (22.3)	440 (24.8)	453 (25.5)	501 (28.3)
*College Graduate or above*	274 (15.5)	351 (19.8)	375 (21.2)	339 (19.1)
***Marital status (%)***				
*Lives alone*	556 (32.3)	550 (32.3)	583 (34.8)	656 (39.9)
*Lives with a partner*	1166 (67.7)	1153 (67.7)	1094 (65.2)	990 (60.1)
***PIR***	2.25 [1.19, 4.19]	2.51 [1.32, 4.39]	2.51 [1.25, 4.45]	2.43 [1.20, 4.32]
***Height (cm)***	167.1 (10.2)	167.4 (10.1)	167.1 (10.1)	168.0 (9.9)
***BMI (kg/m^2^)***	28.75 (5.94)	28.55 (6.40)	28.48 (6.20)	27.99 (6.23)
***Waist circumference (cm)***	99.8 (14.9)	97.8 (15.1)	96.5 (15.3)	94.7 (15.1)
***Systolic blood pressure (mmHg)***	132 (22)	125 (20)	123 (19)	120 (18)
***Diastolic blood pressure (mmHg)***	71 (16)	72 (13)	71 (13)	71 (13)
Active smoking ((cigarettes/day)*years)	0 [0, 0]	0 [0, 0]	0 [0, 0]	0 [0, 0]
***Passive smoking (cigarettes/day)***	0 [0, 0]	0 [0, 0]	0 [0, 0]	0 [0, 0]
***PA level (%)***				
*1*	493 (27.8)	416 (23.5)	422 (23.8)	376 (21.2)
*2*	953 (53.8)	957 (54.0)	921 (51.9)	935 (52.8)
*3*	233 (13.1)	277 (15.6)	317 (17.9)	316 (17.8)
*4*	93 (5.2)	123 (6.9)	113 (6.4)	144 (8.1)
PA MET (MET*minutes*frequency)	0 [0, 327]	60 [0, 429]	68 [0, 480]	105 [0, 551]

Abbreviations: MET, metabolic equivalent of task; PA, physical activity; PIR, poverty to income ratio; Q, quartile.

^1^ Continuous variables are expressed as mean (SD) or median [Q1, Q3]. Categorical variables are expressed as counts (%). In the left column, in parenthesis, the units of measure are reported; this does not apply to pure numbers

**Table 2:** Laboratory variables of study participants, stratified by telomere length quartiles.^1^

	Q1	Q2	Q3	Q4
*n*	1774	1774	1774	1774
***Telomere length (T/S ratio)***	0.74 (0.08)	0.93 (0.04)	1.08 (0.05)	1.36 (0.16)
***Total cholesterol (mg/dL)***	209 (43)	206 (41)	204 (41)	200 (43)
***HDL (mg/dL)***	51 (16)	51 (16)	52 (16)	52 (15)
***C-reactive protein (mg/dL)***	0.28 [0.13, 0.61]	0.24 [0.10, 0.53]	0.24 [0.09, 0.52]	0.19 [0.07, 0.48]
***gHb (%)***	5.4 [5.2, 5.8]	5.4 [5.1, 5.7]	5.3 [5.1, 5.6]	5.2 [5.0, 5.5]
***Leukocyte count (SI)***	7.3 (2.3)	7.2 (2.1)	7.4 (2.1)	7.2 (2.2)
*Lymphocytes (%)*	29.4 (8.8)	29.4 (8.2)	29.6 (8.3)	30.3 (8.9)
*Monocytes (%)*	8.3 (2.2)	8.1 (2.1)	8.0 (2.2)	7.9 (2.3)
*Neutrophils (%)*	58.7 (9.6)	59.1 (9.2)	59.2 (9.4)	58.5 (10.1)
*Eosinophils (%)*	2.4 [1.6, 3.6]	2.3 [1.5, 3.4]	2.2 [1.4, 3.4]	2.2 [1.3, 3.4]
*Basophils (%)*	0.6 [0.4, 0.8]	0.6 [0.4, 0.8]	0.6 [0.4, 0.8]	0.6 [0.4, 0.8]
***Erythrocyte count (SI)***	4.66 (0.51)	4.71 (0.50)	4.67 (0.53)	4.69 (0.54)
***Hb (g/dL)***	14.3 (1.5)	14.3 (1.5)	14.2 (1.5)	14.1 (1.6)
***Hematocrit (%)***	42.3 (4.5)	42.3 (4.4)	41.9 (4.5)	41.8 (4.7)
***Platelet count (SI)***	261.85 (74.56)	267.99 (70.20)	267.47 (64.33)	269.91 (65.60)
***Iron (μg/dL)***	88 (36)	89 (39)	89 (38)	89 (39)
***TIBC (μg/dL)***	365 (65)	367 (66)	371 (68)	372 (69)
***Transferrin saturation (%)***	24.9 (11.2)	25.1 (11.9)	24.7 (11.6)	24.8 (11.5)
***Ferritin (ng/mL)***	107 [51, 206]	87 [41, 175]	77 [33, 153]	71 [28, 148]
***Folate (ng/mL)***	13.7 [9.6, 19.2]	12.8 [9.2, 18.2]	12.4 [9.0, 17.9]	12.5 [8.8, 17.3]
***Cobalamin (pg/mL)***	462 [345, 622]	461 [348, 610]	457 [351, 607]	463 [345, 600]
***Homocysteine (μmol/L)***	8.61 [6.98, 10.89]	8.01 [6.51, 9.95]	7.43 [6.09, 9.22]	7.18 [5.77, 8.89]
***Methylmalonic acid (μmol/L)***	0.14 [0.10, 0.19]	0.13 [0.10, 0.17]	0.12 [0.10, 0.16]	0.12 [0.09, 0.15]
***Cotinine (ng/mL)***	0.06 [0.04, 2.08]	0.08 [0.04, 14.35]	0.09 [0.04, 9.53]	0.11 [0.04, 29.28]
***γ-tocopherol (μg/dL)***	227.0 [139.9, 323.9]	218.0 [143.5, 311.0]	216.5 [143.0, 300.0]	213.0 [145.3, 292.0]
***Retinyl palmitate (μg/dL)***	1.70 [1.00, 2.90]	1.80 [1.10, 2.80]	1.80 [1.10, 2.90]	1.80 [1.10, 2.95]
***Retinyl stearate (μg/dL)***	0.35 [0.35, 0.35]	0.35 [0.35, 0.35]	0.35 [0.35, 0.35]	0.35 [0.35, 0.35]
***Vitamin A (μg/dL)***	61.8 (20.0)	59.1 (17.9)	58.0 (17.3)	55.9 (16.8)
***Vitamin E (μg/dL)***	1270.9 [983.0, 1706.6]	1196.1 [953.9, 1552.6]	1148.9 [908.3, 1511.4]	1053.7 [863.7, 1372.8]
***Triglycerides (mg/dL)***	138 [93, 193]	127 [89, 190]	122 [84, 177]	109 [75, 170]
***LDL (mg/dL)***	125 (34)	126 (35)	121 (34)	123 (38)
***Glucose (mg/dL)***	99.2 [92.1, 109.8]	97.7 [90.6, 107.3]	95.5 [88.1, 104.7]	92.6 [87.1, 101.0]
***C-peptide (nmol/L)***	0.84 [0.61, 1.16]	0.75 [0.55, 1.04]	0.71 [0.52, 0.99]	0.66 [0.50, 0.95]
***Insulin (μU/mL)***	11.45 [7.72, 17.44]	10.55 [7.14, 16.02]	9.75 [7.01, 15.29]	9.80 [6.86, 14.78]

Abbreviations: gHb, glycated hemoglobin; Hb, hemoglobin; Q, quartile; TIBC, total iron binding capacity.

^1^The variables are all continuous, and they are expressed as mean (SD) or median [Q1, Q3]. In the left column, in parenthesis, the units of measure are reported.

After this first general description, we will go to the results of the analyses conducted via Copula graphical Modelling (CGM), looking specifically at the three age groups.

To report the outcomes of the CGM we proceeded as follows. Firstly, we adopted a certainty threshold equal to 0.95 to discern which links found via CGM are consistent after bootstrapping and are therefore more certain. Secondly, for the main analysis of each age group, we selected the variables that are linked to telomere length with a certainty of 0.95 or higher, and we reported them in the three Table 3. Thirdly, we took these same variables from each sensitivity analysis, and we added them to Table 3. In this way it is possible to visualize how consistent are the links found in the main analyses. Fourthly, in the Supplementary Table 3 we reported the partial correlation of the links shown in the Table 3.

**Table 3:** Young. Certainty related to the main analysis and the sensitivity analyses in the Young group.^1^

	**Main analysis**	**Males**	**Females**	**Mexicans**	**Whites**	**Blacks**	**PIR < Mdn**	**PIR ≥ Mdn**	**BMI < 25**	**25 ≤ BMI < 30**	**BMI ≥ 30**	**NHANES 1999-2000**	**NHANES 2001-2002**	**All study population**
*n*	2623	1126	1497	637	1343	421	1213	1219	987	857	750	1193	1430	7096
**NHANES cycle**	1	0.85	1	0.99	1	0.62	1	1	0.98	0.99	1	-	-	1
**Age**	1	1	1	1	1	0.95	1	0.99	1	0.92	0.99	1	1	1
**PA level**	0.95	0.41	0.99	0.76	0.75	0.04	0.94	0.73	0.64	0.31	0.67	0.98	0.57	0.98
**C-reactive protein**	0.98	0.95	0.72	0.21	0.94	0.21	0.86	0.50	0.43	0.91	0.30	0.96	0.52	1
**Basophils**	1	0.94	0.95	0.42	1	0.11	1	0.87	0.79	0.26	0.97	0.75	1	1
**Erythrocyte count**	0.96	0.90	0.37	0.08	0.44	0	0.30	0.33	0.30	0.51	0.08	0.69	0.32	1
**Hb**	0.96	0.59	0.72	0.64	0.39	0.21	0.82	0.19	0.21	0.18	0.47	0.81	0.21	1
**TIBC**	0.98	0.83	0.95	0.33	1	0.30	0.93	0.46	0.85	0.26	0.42	0.55	0.98	1
**γ-tocopherol**	0.99	0.50	0.99	0.98	0.65	0.17	0.91	0.73	0.69	0.77	0.26	0.99	0.44	1
**Retinyl palmitate**	0.97	0.53	0.92	0.32	0.82	0.49	0.92	0.54	0.92	0.20	0.33	1	0.46	0.99
**Retinyl stearate**	1	0.47	0.99	0.27	0.83	0.36	0.52	0.97	0.80	0.43	0.31	1	0.94	1
**Insulin ^2^**	0.95	0.33	0.42	0.18	0.67	0.02	0.23	0.31	0.62	0.22	0.04	0.51	0.24	0.99
**Dietary fibre ^3^**	0.95	0.92	0.57	0.40	0.76	0.15	0.99	0.20	0.16	0.58	0.44	0.92	0.54	1
**Sodium ^3^**	0.95	0.38	0.74	0.08	0.77	0.37	0.62	0.33	0.36	0.16	0.22	0.46	0.59	0.97
**Caffeine ^3^**	0.99	0.58	1	0.52	0.85	0.80	0.98	0.77	0.74	0.53	0.94	0.96	0.97	1
**PFA 22:5 ^3^**	0.95	0.79	0.39	0.86	0.46	0.10	0.46	0.58	0.88	0.09	0.10	0.29	0.94	0.94

Abbreviations: Hb, hemoglobin; PA, physical activity; PFA 22:5, Docosapentaenoic acid; TIBC, total iron binding capacity.

^1^The name of each row indicates the variables that, in the main analysis, are related with telomere length with a certainty ≥ 0.95. In the first column is reported the certainty of the direct correlations between telomere length and the variables in the main analysis; in the other columns, the same is done for each sensitivity analysis.

In dark blue are highlighted the cells where the correlation is negative, and the certainty is ≥ 0.95; while in light blue the cells where the correlation is negative, and the certainty is < 0.95 and ≥ 0.80. In dark green are highlighted the cells where the correlation is positive, and the certainty is ≥ 0.95; and in light green the cells where the correlation is positive, and the certainty is < 0.95 and ≥ 0.80. The non-colored cells indicate a certainty < 0.80.

^2^“Insulin” is colored in red to indicate that it is a variable measured in half of the sample; the participants belonging to this half were requested to come to the examination after at least 8.5 hours of fasting.

^3^The green color indicates the dietary variables, in order not to confuse them with the other variables.

**Table 3:** Middle. Certainty related to the main analysis and the sensitivity analyses in the Middle group.^1^

	**Main analysis**	**Males**	**Females**	**Mexicans**	**Whites**	**Blacks**	**PIR < Mdn**	**PIR ≥ Mdn**	**BMI < 25**	**25 ≤ BMI < 30**	**BMI ≥ 30**	**NHANES 1999-2000**	**NHANES 2001-2002**	**All study population**
*n*	2210	1133	1077	537	1110	365	1019	1020	581	807	793	952	1258	7096
**NHANES cycle**	1	1	1	0.97	1	0.90	0.97	1	0.98	0.98	1	-	-	1
**Age**	1	1	1	0.99	0.99	0.98	1	1	1	0.99	0.98	1	1	1
**Education level**	0.99	1	0.63	0.75	0.90	0.51	1	0.95	0.61	0.86	0.54	0.72	0.95	1
**C-reactive protein**	0.98	0.95	0.84	0.06	1	0.45	0.69	0.97	0.61	0.98	0.15	0.97	0.87	1
**Basophils**	0.99	0.94	0.32	0.10	0.99	0.08	0.94	0.32	0.38	0.43	0.65	1	0.45	1
**Hb**	1	0.97	0.82	0.73	0.74	0.45	0.98	0.87	0.44	0.98	0.84	1	0.66	1
**TIBC**	1	0.97	0.96	0.47	0.90	0.93	0.78	0.98	0.30	0.92	0.69	0.92	0.99	1
**Folate**	0.98	0.61	0.69	0.47	0.74	0.03	0.77	0.31	0.59	0.14	0.38	0.56	0.89	0.95
**γ-tocopherol**	1	0.93	0.98	0.91	0.98	0.86	0.89	0.97	0.87	0.99	0.58	1	0.75	1
**Retinyl stearate**	0.96	0.46	0.74	0.15	0.91	0.30	0.30	0.82	0.31	0.37	0.20	0.99	0.56	1
**Vitamin A**	0.95	0.77	0.52	0.16	0.86	0.04	0.49	0.83	0.13	0.11	0.91	0.56	0.59	1
**Caffeine ^2^**	0.97	0.57	0.96	0.78	0.88	0.21	0.73	0.81	0.31	0.72	0.62	0.24	0.99	1

Abbreviations: Hb, hemoglobin; TIBC, total iron binding capacity.

^1^The name of each row indicates the variables that, in the main analysis, are related with telomere length with a certainty ≥ 0.95. In the first column is reported the certainty of the direct correlations between telomere length and the variables in the main analysis; in the other columns, the same is done for each sensitivity analysis.

In dark blue are highlighted the cells where the correlation is negative, and the certainty is ≥ 0.95; while in light blue the cells where the correlation is negative, and the certainty is < 0.95 and ≥ 0.80. In dark green are highlighted the cells where the correlation is positive, and the certainty is ≥ 0.95; and in light green the cells where the correlation is positive, and the certainty is < 0.95 and ≥ 0.80. The non-colored cells indicate a certainty < 0.80.

^2^The green color indicates the only dietary variable, in order not to confuse it with the other variables.

**Table 3:** Old. Certainty related to the main analysis and the sensitivity analyses in the Old group.^1^

	**Main analysis**	**Males**	**Females**	**Mexicans**	**Whites**	**Blacks**	**PIR < Mdn**	**PIR ≥ Mdn**	**BMI < 25**	**25 ≤ BMI < 30**	**BMI ≥ 30**	**NHANES 1999-2000**	**NHANES 2001-2002**	**All study population**
***n***	2263	1139	1124	543	1151	394	998	1013	590	865	725	1103	1160	7096
**NHANES cycle**	1	0.98	1	0.91	1	0.03	1	0.47	0.75	0.91	0.88	-	-	1
**Sex**	1	-	-	0.94	0.99	0.04	1	1	0.33	0.82	0.99	0.91	0.99	1
**Age**	1	1	1	1	1	0.95	1	1	1	1	1	1	1	1
**PIR**	0.99	0.88	0.95	0.48	0.73	0.46	0.27	0.57	0.99	0.95	0.41	1	0.42	0.99
**Leukocyte count**	1	0.98	0.91	0.86	1	0.03	0.83	0.90	0.80	0.99	0.14	1	0.83	1
**Basophils**	0.96	0.34	0.87	0.15	0.78	0.65	0.66	0.58	0.11	0.55	0.65	0.96	0.41	1
**Erythrocyte count**	0.99	0.99	0.49	0.09	0.77	0.17	0.25	1	0.09	0.58	0.26	0.73	0.96	1
**TIBC**	0.97	0.75	0.68	0.18	0.80	0.09	0.26	0.87	0.13	0.25	0.18	0.30	0.96	1
**Ferritin**	0.99	0.99	0.21	0.76	0.75	0.07	0.10	1	0.81	0.66	0.34	0.47	1	0.98
**γ-tocopherol**	1	0.89	0.48	0.18	0.83	0.04	0.46	0.27	0.19	0.62	0.66	0.56	0.94	1
**Retinyl stearate**	1	0.93	0.60	0.48	0.58	0.56	0.55	0.78	0.64	0.65	0.35	1	0.80	1
**LDL**	0.97	0.85	0.39	0.16	0.66	0.29	0.86	0.37	0.70	0.15	0.35	0.72	0.62	1
**MFA 20:1 ^2^**	0.97	0.65	0.57	0.51	0.21	0.37	0.28	0.89	0.29	0.20	0.76	0.77	0.46	0.91

Abbreviations: MFA 20:1, Eicosenoic acid; PIR, poverty to income ratio; TIBC, total iron binding capacity.

^1^The name of each row indicates the variables that, in the main analysis, are related with telomere length with a certainty ≥ 0.95. In the first column is reported the certainty of the direct correlations between telomere length and the variables in the main analysis; in the other columns, the same is done for each sensitivity analysis.

In dark blue are highlighted the cells where the correlation is negative, and the certainty is ≥ 0.95; while in light blue the cells where the correlation is negative, and the certainty is < 0.95 and ≥ 0.80. In dark green are highlighted the cells where the correlation is positive, and the certainty is ≥ 0.95; and in light green the cells where the correlation is positive, and the certainty is < 0.95 and ≥ 0.80. The non-colored cells indicate a certainty < 0.80.

^2^The green color indicates the only dietary variable, in order not to confuse it with the other variables.

In order to better understand and discuss the results, we have created Supplementary Tables that will come into play only in the “Discussion” chapter. Supplementary Tables 4–6 display some characteristics of the entire study population after stratification for quartiles of caffeine intake (Supplementary Table 4), dietary fibers intake (Supplementary Table 5), and serum levels of γ-tocopherol (Supplementary Table 6).

Finally, it is important to notice that, when we refer to “tables” and “figures” in the plural form, it means that there are three “copies” of that element: one for each stratum.

### Participants’ characteristics

In Table 1 and in Supplementary Table 1, you can first notice the strong inverse relation between TL and the age of the participants: the higher the age, the shorter the telomeres. Two other clear trends are the increasing percentages of females and blacks, while moving from the lowest to the highest quartiles of TL. A similar increase is also seen in the education level, where the percentage of people with a high-school degree or higher is more present in the highest quartiles of TL.

There are three more characteristics that are worth mentioning: the first is that people with shorter telomeres belong predominantly to the 1999-2000 NHANES cycle, where participants are slightly older than in the 2001-2002 cycle. The second is that the higher the quartile of TL, the more people are physically active, and the lower their waist circumference is. The third is the absence of any trend for active and passive smoking.

All these trends (or absence of trends) are consistent throughout the entire study population and the age stratifications.

Differently from what we have just seen, the trends that are visible in Table 2 for the entire population are much more blunted in the three age groups (Supplementary Table 2). The first relation that you can find consistently in the table is the one between TL and the marker of inflammation C-reactive protein (CRP): the longer telomeres are, the lower the blood levels of CRP. Then, telomere length is higher when blood levels of vitamin A, vitamin E and γ-tocopherol are lower. Yet, this is not true for all the age groups: the trend for vitamin A is not present in any of the single strata, while the trend for γ-tocopherol remains, but it is blunted; lastly, while less vitamin E is related with longer telomeres in the Young group, this is not the case for the Middle, and the trend is even inverted in the Old group.

### CGM’s outcomes

Before looking in detail at the outcomes of the Copula Graphical Modelling (CGM), you might notice in the three Table 3 that the main analyses of the three age groups have some links in common: among the 16 certain links of the Young group, 9 overlaps with the Middle (12 certain links in total), and 7 with the Old (13 certain links in total); while the Middle and the Old group share only 6 links. Actually, these 6 links are also shared with the Young group, therefore they are independent of age stratification. It is also worth noting that the higher the number of participants in an age group, the higher the number of links that are certain due to higher statistical power; so, the Young group is the first group for number of participants and for links that are certain; then there is the Old group; and, finally, the Middle group.

Now we are going to describe certain links that we deem to be the most interesting and relevant. These links are all reported in the three Table 3.

As previously anticipated, longer telomeres are associated with younger age, with the NHANES cycle 2001-2002 and with lower serum levels of γ-tocopherol. These are the only three findings that maintain a certainty ≥ 0.95 in all the age groups and, at the same time, in a big part of their sensitivity analyses. Moreover, these are also the only three findings that are clearly consistent with the trends we have described so far (Tables 1, 2 and Supplementary Tables 1, 2).

It is then interesting to also look at the less consistent links, and, more broadly, at the links we have not found. In the first place, blood levels of CRP are inversely related to TL in the Young and Middle group, but they do not reach certainty threshold in the Old group. Besides, neither black race nor female sex is linked to longer telomeres after correcting for all the other variables, except in the Old group, where being female (but not black) is related to longer telomeres. Moreover, links between TL and indicators of socio-economic status, physical activity and metabolic syndrome are almost completely absent. Finally, BMI, waist circumference, active and passive smoking, serum cotinine levels and energy intake are not directly connected with TL in our analyses.

At this point, we turn our focus to the last meaningful relationships, which are the ones between TL and dietary variables. These variables were not shown in the previous tables, therefore no paragon will be done with previous findings.

The most striking fact is that, despite around 50 dietary variables being included in the analyses, they are almost completely absent from the CGM’s outcomes: only one fatty acid in the Old group, only caffeine in the Middle and Young group, and only three other dietary variables in the Young group. Among these variables it is interesting to see that, in the Young group, sodium consumption is related to longer telomeres, while dietary fibers to shorter telomeres; and also, both in the Young and Middle group, caffeine is negatively related to TL.

Looking at the tables containing the results, you might notice that the threshold of 0.95 of certainty is seldomly reached in the sensitivity analyses. This is true, but there is one last thing we should consider to better comprehend the actual consistency of the results obtained throughout the sensitivity analyses: the sign of the links. If you look at the three Supplementary Table 3 line by line, variable by variable, you can notice that every link either maintains the same sign or becomes zero, but it does not change from positive to negative, or vice versa. The only two changes of sign are: the serum retinyl palmitate for the analysis of the Young overweight adults, and the serum retinyl stearate for the NHANES cycle 2001-2002 in every age group.

To conclude, except the serum retinyl palmitate and the serum retinyl stearate, all the links discovered in the main analyses are quite solid, even when they do not reach the certainty threshold in the sensitivity analyses. Still, the strongest connections of all are the negative link between TL and age, and TL and serum γ-tocopherol, and the link between TL and the NHANES cycle 2001-2002.

## DISCUSSION

The goal of our study was to capture the conditional dependence relationship between telomere length (TL) and demographic, lifestyle and dietary factors by using Copula Graphical Models (CGMs) on NHANES 1999-2002.

Even though, by definition, a data-driven exploratory study does not have any hypothesis, the root idea behind our study was that human nutrition could exert a direct influence on leukocyte telomere length (LTL). This idea has been strongly contradicted by the results of the analyses. Indeed, in our analyses only 5 dietary variables out of 50 have been directly related to TL with a certainty ≥ 0.95 after correcting for the effect of all the other variables in the dataset. These variables are the following: caffeine and dietary fibers, related to shorter telomeres; eicosenoic acid, docosapentaenoic acid and sodium, related to longer telomeres. However, these relationships reach the certainty threshold of 0.95 only in specific age strata.

The factors that are mostly linked to TL, except for age, are blood variables. Among these variables there are at least six that could be influenced by diet, and they are: the blood levels of C-reactive protein (CRP), the serum levels of retinyl stearate and γ-tocopherol, that all have a negative connection to TL; the serum levels of retinyl palmitate, folate and vitamin A, that have a positive connection to TL.

Even though only a small part of the links found in the main analyses reaches the certainty threshold throughout the sensitivity analyses, almost all the connections maintain the same direction, suggesting that the results of our main analyses are consistent. Therefore, for the sake of simplicity, we will not highlight further the differences and commonalities between the main analyses and the sensitivity analyses, unless strictly necessary; and when referring to the analyses, we will implicitly refer to the three main analyses.

If not otherwise specified, the trends and connections reported are all taken from the three Table 3.

### Interpretation and comparison with literature

We are first going to discuss the dietary variables, then we will look at the blood variables, and finally we will conclude with the sociodemographic and lifestyle variables.

The negative relation we have found between caffeine and TL is present with a certainty ≥ 0.95 in the Young and Middle group, and therefore it is the most consistent among the diet-TL links. This negative relation is not totally unexpected, since Tucker in 2017 [35] performed a linear regression analysis and showed that in NHANES there is a negative correlation between TL and caffeine, but a positive one between TL and coffee consumption. However, a study based on the Nurses’ Health Study and published by Liu et al. in 2016 [36], found that coffee consumption is positively related to the length of telomeres, and caffeine is positive, but not significantly, related to TL after adjusting for coffee consumption. A possible explanation for these differences is a sampling bias in NHANES. In fact, the caffeine intake is marginally related to higher energy intake, higher serum cotinine levels, and active and passive smoking (Supplementary Table 4). These relations suggest that in NHANES 1999-2002 high caffeine consumption goes hand in hand with some poor health choices; and therefore, the negative correlation between caffeine and TL might be caused by the specific sample selected in the study.

We have also found two fatty acids that are positively related to TL: eicosenoic acid and docosapentaenoic acid. However, these two names refer to different types of fatty acids, from ω-3 to ω-11, making it not possible to understand an eventual mechanism of action on leukocyte telomere length. Moreover, this outcome is not consistent in the analyses, and it does not reach the certainty threshold in the sensitivity analysis with the entire study population, which is the one with the most statistical power. So, it seems more likely that this result is an artifact due to the inclusion of 19 different types of fatty acids in our study.

We can apply a similar reasoning to the positive partial correlation between sodium intake and TL found only in Young, because, while the link is positive and above certainty in Young, it is not certain in Middle, and negative, but not certain, in the Old. Therefore, it is quite inconsistent.

The last queer diet-TL relation is the negative correlation between dietary fibers and TL. In this case, however, the link is more consistent than for the fatty acids and the sodium: the negative link we have found in the Young remains negative in the other groups, even if it does not reach the certainty threshold. Contrary to us, Tucker (2018) found that, the higher the consumption of fibers, the longer the telomeres in NHANES [37]. Not only, but there is an abundant number of studies that show the positive effects of a diet rich in fibers on the length of telomeres [20, 25–27, 38]. Due to these previous findings of other researchers, we cannot explain the negative association fibers-TL as an inverse causation.

To elucidate the negative correlation between fibers and TL, it might be important to consider the source of the dietary fiber and the related quantity of food consumed. In Supplementary Table 5, higher consumption of fibers is strongly marginally correlated with higher energy intake. This finding suggests that the high quantity of food may be the main source of fibers of the participants with high fiber intake. Indeed, this hypothesis can explain our results, but it does not clarify the divergence with another research in NHANES. To solve this last problem, we can look at the exclusion criteria of the participants: we put 10000 kcal as upper limit of daily caloric intake, while other studies are much more restrictive [20, 37]. This difference in the exclusion criteria might affect the ultimate result. In fact, high intake of dietary fibers might be tightly connected to overeating and poor health in our population, but not in the populations of studies where the maximum energy intake is lower compared to ours.

Concerning the serum nutrient levels, we can first notice that there seem to be connections between vitamin A-related compounds and TL. However, these connections are mixed: vitamin A is associated with longer telomeres in the Middle and Old group, but it reaches the certainty threshold only in the Middle. Retinyl stearate has a positive partial correlation with TL, but the link is not robust. Finally, retinyl palmitate is linked to shorter telomeres for Young and Middle, but it is certain only for the Young. Therefore, the connection between vitamin A and TL is unclear and might not be present. The silver lining is that there is a lack of studies concerning vitamin A, retinyl stearate, retinyl palmitate and their effects on telomeres, and this is an opportunity for new research.

The serum levels of folate encounter the same issue of consistency that we have found for the dietary intake of fatty acids: the relation is present and certain only for the Middle group, but it disappears almost entirely for the other two groups. So, this association is too weak to be confident that it is real.

With regard to serum levels of vitamin E (α-tocopherol) and γ-tocopherol, the former does not influence the telomeres, while the latter has a robust negative association with TL. It is peculiar to see such a different behavior in two nutrients that are so similar. Nevertheless, this is not totally unexpected, because Tucker (2017) obtained the same results in NHANES [39]. It seems therefore clear that serum vitamin E does not influence LTL, while γ-tocopherol is associated with decreased TL.

Serum γ-tocopherol has been linked to mixed health outcomes, making it not totally comprehensible how it exerts its effects in the human body [40, 41]. As other researchers have proposed [42], we believe that the serum levels of γ-tocopherol might be related to poor health. Indeed, there are complex mechanisms that regulate lipid-soluble vitamins, and the ones underlying γ-tocopherol are not fully understood yet [42, 43]. What we can see from our data is that γ-tocopherol has a positive marginal correlation with BMI, waist circumference, blood levels of total cholesterol and glycated hemoglobin (Supplementary Table 6), all indicators of poor diet and lifestyle. These marginal correlations suggest that γ-tocopherol might be maintained in higher levels in the bloodstream especially in people in poor health conditions.

One last remark to conclude the discussion on serum α- and γ-tocopherol: the absence of link between the α-tocopherol and TL, and the negative link between γ-tocopherol and TL suggest that these two potent antioxidants do not preserve telomere length. Therefore, oxidative stress might not be a factor of telomere shortening *in vivo*.

Last, and maybe most important, we now consider the negative direct association between blood levels of C-reactive protein (CRP) and TL. C-reactive protein is a well-known marker of inflammation [44], which in turn might be deleterious for the telomeres. Indeed, the direct association CRP-TL suggests that inflammation can accelerate the process of telomere attrition, as previously reported by other authors [29, 30]. Yet, most importantly, it gives a hint on how diet and other factors can influence TL: CRP might mediate the effects that some factors have on TL, as we will show further in this discussion. So, CRP and inflammation might act as mediators between human behaviors and human telomere length.

We were expecting to find some strong connections between telomere length and the race, sex, education level, poverty to income ratio (PIR) of the participants, as other researchers did before us [18, 45, 46]. However, it appears that, after correcting other factors, these associations fade. We have found no connection between race and TL, despite the fact that African Americans have longer telomeres compared to Caucasian in raw data (Table 1) and in literature [45, 46]. So, probably the differences between LTL among races are linked to other factors, like leukocyte composition [31]. We can apply an analogous logic to sex: in our analysis females have longer telomeres than males only in the Old group. Therefore, the results are not robust. In this specific case, however, we believe that this finding supports an idea expressed by Okuda et al. in 2002: the differences in LTL between sexes arises slowly in time due to a lower attrition in females compared to males [3]. This same “slow effect” might be the answer to why we see that PIR has a negative correlation with telomeres only in the Old group, and not in the others.

Conversely, the higher the level of education, the longer the telomeres for the Young and Middle; and the certainty is ≥ 0.95 only in the Middle. Higher education is generally related to higher wealth and a better lifestyle, and this might be the reason why we see this connection in the two younger groups. As for the Old group, since we are looking at a sample of people selected between 1999 and 2002, it is important to keep in mind that only a few of the older participants could have had the privilege of being highly educated; therefore this index might simply lose power on the Old.

We also expected some clear relations between physical activity, BMI, waist circumference, smoking status and TL, since they are present in the literature [19, 22, 23]. But even in this case we were surprised by the absence of links. Of these four factors, only physical activity (PA) has a positive effect on TL; more specifically, only the level of PA assessed in a scale from 1 to 4 has this relation and only in the Young group; therefore, we cannot conclude that being physically active is directly related to TL.

Once again, Tucker in 2017 [22] studied the relationship between TL and PA in NHANES, and he discovered that being more physically active is protective for the telomeres. So, probably this effect is mediated by other factors that were not considered in previous studies. In fact, physical activity shows a negative partial correlation with CRP, and it is also inversely related to BMI and waist circumference with certainty ≥ 0.95, which in turns have a positive partial correlation with CRP with certainty ≥ 0.95. The same positive partial correlation is found for active smoking and CRP, again with a certainty above the threshold.

Therefore, we hypothesize that C-reactive protein plays an important role as a mediator between these lifestyle factors and telomere length, and it should always be considered as a confounder.

### Strengths and weaknesses

The first strength of our study is the fact that we conducted the analysis in a data-driven way, therefore almost no assumptions were made *a priori*. This implies that we could add a relatively large number of variables to the model (p = 102) without doing a strict selection; and so, we included a remarkably higher number of variables in comparison with studies that use other methods on NHANES data [18, 28, 30]. On the other hand, we had to make a loose selection of the variables anyway. We decided to include only the variables that could be related to LTL and that were potentially present for all the participants (so, for example, we excluded pregnancy status). Therefore, even if our study is data-driven, it still has a certain degree of subjectivity.

Another strength is that we could include missing values in our analyses. So, while in other studies a non-trivial number of participants is excluded because of the presence of missing values [22, 24, 28], we included the vast majority of NHANES participants with TL data (7096 out of 7839). On the one hand, this inclusion of more participants increased the statistical power of the analyses; but on the other hand, it might have had a negative impact on some of the outcomes of CGMs, because of the method we used to identify the copula: the nonparanormal shrinkage (npn). Indeed, the npn is not optimal in the presence of missing values; and alternative methods for copula estimation, like gibbs sampling, perform better in such situations [33, 34].

A third strength is that we divided the sample in three age groups, in order to obtain more specific and accurate results. In this way we were able to clearly see the effects of some factors on TL only in specific strata, and we could formulate hypothesis consequently.

Obviously, this study shares the limitation of all the cross-sectional studies that involve LTL assessment via qPCR. The inability to infer causality typical of cross-sectional studies can be delicate in our case, because, of course, you do not expect that people with shorter telomeres will choose a specific type of food only because they have short telomeres; nevertheless, sometimes reverse causation might be possible. Moreover, it is not possible to know the direction of the relations between TL and hemoglobin, total iron binding capacity (TIBC), percentage of basophils and erythrocyte count, and whether these are just characteristics that accompany longer (or shorter) telomeres. As for the qPCR method developed by Cawthon in 2002, it is a ground-breaking instrument to measure the telomere length of a vast number of people, because it is cheap, fast, and it does not need highly-specialized personnel; however, it is not as accurate and precise as the gold standard, which is electrophoresis in agarose gel; and this feature leads to higher uncertainty even in the measure of telomere length, that is the most valuable variable of the present study [47].

Finally, in the past decades researchers discovered that LTL can reflect good health and healthy ageing or poor health and premature ageing; however, already in 1994, Slagboom et al. proved that TL has a high heritability [15]. This implies that, also for future research, it would be better to use leukocyte telomere length in studies that plan a series of measurements over time on the same participants, such as longitudinal studies or clinical trials. In this way it is possible to use the relative telomere attrition as the main outcome.

Conversely, these last shortcomings of our study imply a hidden strength: since a big portion of telomere length is explained by genetics, and a part is explained by the use of qPCR, the few links that we have obtained must be strong enough to overcome the effect of genetics and measurement-related uncertainty.

## CONCLUSIONS

Our study shows that nutrients and other factors that are commonly known to impact telomere length have little to no direct association with it. This means that probably these factors exert their effect on leukocyte telomere length in an indirect way; and C-reactive protein is a potential central mediator for this.

The central role played by CRP and the marginal role of antioxidants suggests that telomeres are particularly vulnerable not to oxidative stress, but to inflammation; and they should be protected against it. However, to be more confident about the results obtained, it would be important to apply Copula Graphical Modelling on other large study populations, and it would also be important to estimate the copula using a method that includes more iterations, such as gibbs sampling, instead of a simple transformation, such as nonparanormal. Lastly, for some of the unexpected links that we have found there is a logical next step: performing a regression analysis to obtain the magnitude of the effect.

## MATERIALS AND METHODS

### Study population

The National Health and Nutrition Survey (NHANES) is a cross-sectional survey conducted in the U.S.A. since the 1960’s by the National Center for Health Statistics (NCHS), a section of the Centers for Disease Control and Prevention (CDC). The data obtained via this survey include demographic data, medical examinations, laboratory tests, lifestyle and dietary habits. All these data are publicly available online without the payment of any fee.

Since 1999 NHANES has been conducted on a yearly basis and is divided into blocks of 2-years cycles. Each year a sample of around 7000 people is selected for the survey, and around 5000 people complete the examinations and interviews. The sampling procedure implemented consists of a four-stage probability sampling design, and it is done in order to obtain samples that are representative of the non-institutionalized U.S. population. Further information on sampling is available at NHANES website [48].

Our analysis was conducted on two NHANES cycles: the cycles of 1999-2000 and 2001-2002. This choice was made because these are the only two cycles with available data of telomere length (TL). In the years 1999-2002, a total of 7839 adults aged 20 and older provided blood samples and informed consent for DNA analysis; therefore, the total sample of NHANES participants that could be analyzed consisted of 7839 adults. However, people aged 85 and older were defined as “85+” in NHANES datasets (*n* = 225), and they were excluded from our analysis, since the exact age is an important factor. Then, outliers were removed to increase the quality of the analysis. In particular, people with highly implausible caloric intake were excluded from the study. The thresholds were set at <500 kcal/day for females, <800 kcal/day for males (*n* = 430), and >10000 kcal/day for both sexes [49]. Also, a few people with a value of telomere length coded as “NA” were excluded (*n* = 9). Finally, after log-transforming TL, the participants with telomere length that was an outlier value were discarded (*n* = 75). Telomere length values were defined as outliers if they were lower than the difference between the 1^st^ quartile and 1.5 the interquartile range (IQR), and higher than the sum between the 3^rd^ quartile and 1.5 IQR.

The final study population was 7096 participants aged 20-84 with available telomere length data.

**Figure 1:**
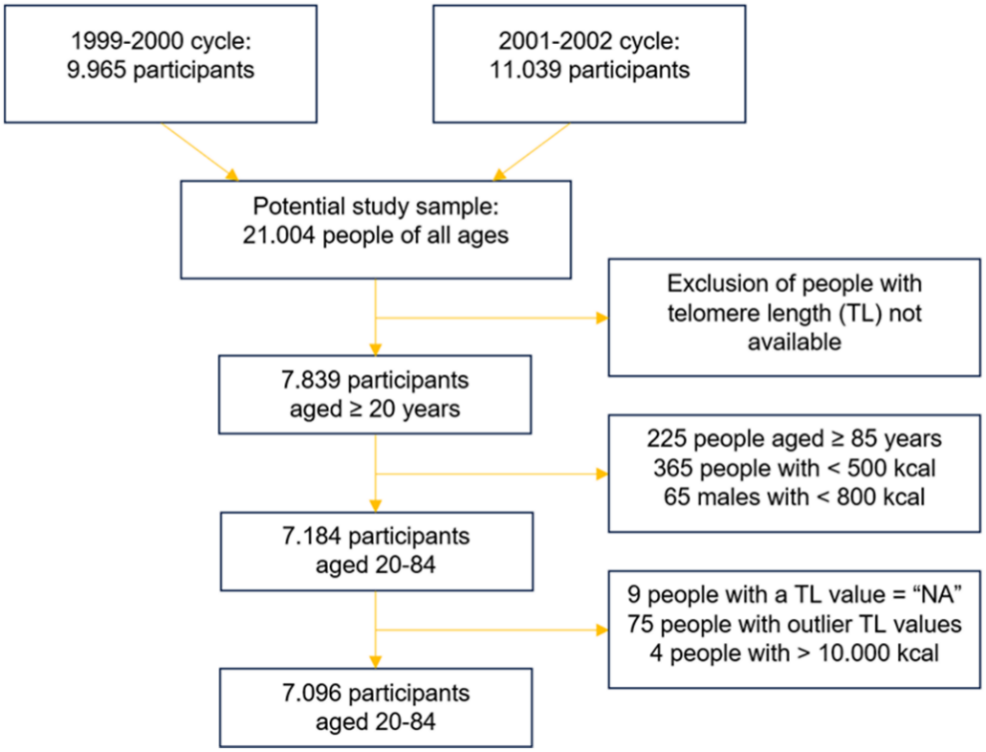
Merging and skimming of the original NHANES datasets.

An overview of this process is given in Figure 1.

The final study population was then split into three age groups: Young from age 20 to 39 (*n* = 2623), Middle from 40 to 59 (*n* = 2210), and Old from 60 to 84 (*n* = 2263). This choice was made to obtain more accurate and specific results.

### Variables

### Telomere length measurement

Telomere length was measured on leukocytes. Hence, in NHANES the data referring to TL are in fact leukocyte telomere length (LTL). The reason underlying this choice is that blood cells are available without the use of invasive procedures and, at the same time, LTL is considered to be a biomarker of ageing and a proxy of TL of replicating cells [18, 50].

Leukocyte telomere length was measured via quantitative polymerase chain reaction (qPCR) by the laboratory of Elizabeth Blackburn at the University of California, San Francisco, following the protocol developed by Cawthon in 2002 [51]. In the end LTL was expressed as T/S ratio, which is telomere length relative to the reference gene of human beta-globin. More details of the procedures were reported by the group that performed this assay on the NHANES samples [18].

### Demographic variables

During each NHANES survey, every participant completed a self-administered questionnaire containing questions about demographic information.

Several demographic variables were considered in our analysis in order to include a high number of potential confounders, covariates and mediators. Some of these variables were included directly by using the values coded by NHANES; this was the case for sex, age, education level and poverty to income ratio (PIR). In NHANES, sex is a discrete variable coded as male = 1, female = 2; age is a continuous variable from 20 to 84 years old; education level is a discrete variable from 1 to 5, where 1 is the lowest education level and 5 is the highest; finally, PIR is a continuous variable from 0 to 5, while PIR ≥ 5 is coded as 5.

For the other demographic variables, it was necessary to apply slight modifications. These variables are race and marital status. In NHANES survey, race is coded as Mexican American, Other Hispanic, Non-Hispanic White, Non-Hispanic Black and Other Race with a crescent value from Mexican American = 1 to Other Race = 5; however, in our analysis we included one dummy variable per each race, in order to better study the relation between race differences and TL. Marital status was also included as a dummy variable; in this case a value of 0 corresponded to living alone (divorced, widowed, separated, never married), while 1 was the opposite (married, living with a partner).

One last dummy variable was included to specify the participants’ belonging to the 1999-2000 or 2001-2002 cycle of NHANES.

### Lifestyle variables

Every participant was asked to complete another self-administered questionnaire. The topic of this second questionnaire concerned lifestyle-related behaviors.

Two main lifestyle variables were included in our study: physical activity and tobacco exposure. Physical activity (PA) was calculated in two ways. Firstly, as reported average PA with discrete values from 1 to 4, where 1 is the lowest average PA and 4 is the highest. Then, by multiplying the frequency of a type of PA done by the participant in the past 30 days per its average duration and its metabolic equivalent of task (MET), and by diving it all by 100, to obtain more manageable numbers.

Tobacco exposure was calculated in 3 ways. First, as the product of average cigarettes smoked by the participant per day and number of years in which the participant smoked that amount. Second, passive smoking exposure was calculated as cigarettes smoked on average inside the house where the participant lived. Third, tobacco active and passive exposure was considered as serum cotinine levels (that is a laboratory variable). Due to the lack of data regarding the grams of tobacco consumed in forms that are different from cigarettes (cigars, pipes, etc.), only cigarettes are considered for the active and passive smoking exposure.

### Examination variables

The examination variables were all measured by an expert in a mobile examination center (MEC), which is the unit where NHANES medical examinations and blood drawings are performed. In our analysis, we included the measures of systolic and diastolic blood pressure (mmHg), height (cm), BMI (kg/m^2^) and waist circumference (cm). These few variables are important because they can reflect the health status of the participants and, in the case of blood pressure, the presence or absence of hypertension.

### Laboratory variables

Laboratory variables, including telomere length and serum cotinine, were obtained by analyzing venous blood samples that were drawn by the MEC staff in the MEC facility. The samples were stored at -20° C and then transported to different laboratories for the subsequent analyses. However, this is not the case for the blood cell count, because it was conducted in the MEC laboratory itself.

For our analysis we considered white blood cell count (SI) and composition (%), red cell count (SI), hemoglobin (g/dL), hematocrit (%), platelet count (%), C-reactive protein (mg/dL), total cholesterol and HDL (mg/dL), glycated hemoglobin (%). We also included iron (μg/dL), total iron binding capacity (μg/dL), transferrin saturation (%), folate (ng/mL), vitamin B_12_ (pg/mL), homocysteine (μmol/L), ferritin (ng/mL), methylmalonic acid (μmol/L), γ-tocopherol (μg/dL), retinyl palmitate (μg/dL), retinyl stearate (μg/dL), retinol (μg/dL) and vitamin E (μg/dL).

In both NHANES cycles, a random half of the participants was selected to undergo a fasting blood examination in the morning, with a minimum fasting period of 8.5 hours. The variables previously reported were common between the fasting group and the non-fasting group. However, the fasting group had also some unique variables. Among these “fasting variables” there were some that we included in the analysis, namely: LDL cholesterol (mg/dL), triglycerides (mg/dL), plasma fasting glucose (mg/dL), C-peptide (mmol/L) and insulin (μU/mL).

### Dietary variables

The diet was assessed via a 24-hour dietary recall interview conducted by an expert. During the interview, participants tried to remember food and food quantities consumed in the preceding 24 hours. The estimates of the total food consumed were then recorded on a computer and are now available as “individual foods file”. The data of the individual foods file was then translated into food components using the United States Department of Agriculture (USDA) Survey Nutrient Database. Therefore, it is possible to have access to macro- and micronutrients consumed by the participants, and to the estimated daily caloric intake.

In our analysis we used only the data concerning food components. For the sake of simplicity, all the dietary variables used in the current study are not reported here. Nevertheless, it is possible to display the variables in their entirety by accessing the link of the study preregistration [52].

### Data analysis

Prior to the analysis, all the variables were standardized. The only exceptions were TL, age, PIR and the categorical and dummy variables.

The steps that are described in the following paragraphs were performed in each one of the three age groups (Young, Middle and Old), even if it is not explicitly stated.

Preregistration of the analysis was performed [52].

### Copula Graphical Modelling

The statistical analysis was done using Copula Graphical Models (CGMs). Particularly, Gaussian Copula graphical models measure the partial correlations among all the observed variables. The partial correlation between two variables, as opposed to the marginal correlation, is the correlation that the variables have after considering the effect of all the other variables at stake. In other words, the partial correlation is a direct association, and the variables with a partial correlation are conditionally dependent on each other, while the variables without a partial correlation are conditionally independent to each other, given the remaining variables.

The output of an analysis where CGMs are exploited takes the form of an undirected graph constituted by vertices and edges. Each vertex of the graph represents a variable included in the model. While each edge that connects two vertices represents the conditional dependence between the vertices. According to the pairwise Markov property, a pair of vertices that are not connected by an edge are “pairwise conditionally independent”.

### Nutrinetwork function

The software R (version 4.3.1) was used for the entire data analysis. More specifically, the R package nutriNetwork was exploited to apply CGMs to the dataset. The main function of this package is also called nutriNetwork, and it was used mainly with the default options [34]. However, the method used was method = “npn”, instead of the default method = “gibbs”; moreover, the penalty parameter *rho* was put equal to a vector of length 50 that ranged from a maximum value of 0.1 to a minimum of 0.001.

### Selectnet function

The function selectnet from the nutriNetwork package was then used to select the optimal graph among the 50 generated with nutriNetwork (one graph for each rho penalty). selectnet was run with all the default options. This means that the criterion used for the model selection was the extended Bayesian Information Criterion (eBIC), which has previously shown to perform well in cases like ours [34, 53].

### Certainty analysis

Nonparametric bootstrapping was done to assess the underlying uncertainty related to the estimated edges. This resampling procedure was applied 100 times, which entails that the analysis previously described (nutriNetwork + selectnet) was repeated on various computer-generated samples. The more the relation between two variables is certain, the more times this relation will appear throughout the resampling analysis. A value above 0.95 was considered certain. 0.95 means that the direct association between two variables was found in 95 out of 100 resampled datasets.

### Sensitivity analysis

To test the robustness of the models, a last step was taken: a sensitivity analysis.

The sensitivity analysis makes it possible to see whether the results obtained on the whole dataset are also applicable to certain categories. In our specific case, the model ran once per each of the following stratifications: Males, Females, Mexican Americans, Whites, Blacks, PIR < Mdn PIR, PIR ≥ Mdn PIR, BMI < 25kg/m^2^, 25kg/m^2^ ≤ BMI < 30kg/m^2^, BMI ≥ 30kg/m^2^, 1999-2000 NHANES cycle, 2001-2002 NHANES cycle. This means that the model ran only on male participants, then only on females, Mexican Americans, and so on. The last sensitivity analysis was performed by applying CGMs on the entire study sample (*n* = 7096).

## Supplementary Material

Supplementary Tables 1 and 2

Supplementary Table 3

Supplementary Tables 4-6
