# Supplementary material for: Diet, lifestyle and telomere length: using Copula Graphical Models on NHANES data
Source: Aging (Albany NY). 2025 Jan 29;17(2):329–56. doi: 10.18632/aging.206194 (PMC11892917; doi:10.18632/aging.206194)
Supplement: Supplementary Tables 1 and 2 [file aging-17-206194-s001.pdf]

SUPPLEMENTARY TABLES

Supplementary Table 1. Young. Demographic, examination and lifestyle characteristics of the Young group, stratified by telomere length quartiles.[1]

	Q1	Q2	Q3	Q4
n	656	656	656	655
Telomere length (T/S ratio)	0.85 (0.08)	1.04 (0.04)	1.19 (0.05)	1.45 (0.15)
NHANES cycle (%)				
1999-2000	367 (55.9)	287 (43.8)	267 (40.7)	272 (41.5)
2001-2002	289 (44.1)	369 (56.2)	389 (59.3)	383 (58.5)
Sex (%)				
Female	361 (55.0)	395 (60.2)	369 (56.2)	372 (56.8)
Male	295 (45.0)	261 (39.8)	287 (43.8)	283 (43.2)
Age (years)	30 (6)	30 (6)	29 (6)	28 (6)
Race (%)				
Mexican American	214 (32.6)	189 (28.8)	179 (27.3)	139 (21.2)
Other Hispanic	41 (6.2)	42 (6.4)	36 (5.5)	61 (9.3)
White	288 (43.9)	308 (47.0)	292 (44.5)	286 (43.7)
Black	90 (13.7)	89 (13.6)	120 (18.3)	143 (21.8)
Other	23 (3.5)	28 (4.3)	29 (4.4)	26 (4.0)
Education level (%)				
Less Than 9th Grade	71 (10.9)	50 (7.6)	67 (10.2)	53 (8.1)
9-11th Grade	142 (21.7)	103 (15.7)	130 (19.8)	111 (17.0)
High School Graduate	160 (24.5)	160 (24.4)	150 (22.9)	179 (27.4)
Some College or AA degree	173 (26.5)	203 (31.0)	185 (28.2)	199 (30.4)
College Graduate or above	108 (16.5)	139 (21.2)	123 (18.8)	112 (17.1)
Marital status (%)				
Lives alone	197 (31.3)	246 (39.2)	265 (42.7)	272 (44.7)
Lives with a partner	432 (68.7)	382 (60.8)	356 (57.3)	336 (55.3)
PIR	2.07 [1.08, 3.72]	2.36 [1.24, 3.99]	2.13 [1.09, 3.94]	2.04 [0.99, 3.82]
Height (cm)	167.8 (10.4)	167.4 (9.6)	167.8 (10.1)	168.0 (9.8)
BMI (kg/m²)	28.24 (6.52)	28.16 (6.19)	27.44 (6.23)	27.36 (6.41)
Waist circumference (cm)	94.8 (16.3)	94.1 (15.0)	93.1 (16.0)	91.8 (14.8)
Systolic blood pressure (mmHg)	114 (12)	114 (12)	113 (12)	113 (12)
Diastolic blood pressure (mmHg)	69 (13)	70 (12)	68 (12)	68 (13)
Active smoking ((cigarettes/day)*years)	0 [0, 0]	0 [0, 0]	0 [0, 0]	0 [0, 0]
Passive smoking (cigarettes/day)	0 [0, 0]	0 [0, 0]	0 [0, 0]	0 [0, 0]
PA level (%)				
1	151 (23.0)	153 (23.3)	137 (20.9)	123 (18.8)
2	337 (51.4)	312 (47.6)	325 (49.5)	335 (51.1)
3	99 (15.1)	140 (21.3)	128 (19.5)	140 (21.4)
4	69 (10.5)	51 (7.8)	66 (10.1)	57 (8.7)
PA MET (MET*minutes*frequency)	72 [0, 432]	107 [0, 513]	123 [0, 650]	114 [0, 552]

Abbreviations: MET, metabolic equivalent of task; PA, physical activity; PIR, poverty to income ratio; Q, quartile.
[1]Continuous variables are expressed as mean (SD) or median [Q1, Q3]. Categorical variables are expressed as counts (%). In the left column, in parenthesis, the units of measure are reported; this does not apply to pure numbers.

Supplementary Table 1. Middle. Demographic, examination and lifestyle characteristics of the Middle group, stratified by telomere length quartiles.[1]

	Q1	Q2	Q3	Q4
n	553	553	552	552
Telomere length (T/S ratio)	0.76 (0.08)	0.93 (0.04)	1.07 (0.04)	1.34 (0.15)
NHANES cycle (%)				
1999-2000	300 (54.2)	228 (41.2)	214 (38.8)	210 (38.0)
2001-2002	253 (45.8)	325 (58.8)	338 (61.2)	342 (62.0)
Sex (%)				
Female	244 (44.1)	268 (48.5)	285 (51.6)	280 (50.7)
Male	309 (55.9)	285 (51.5)	267 (48.4)	272 (49.3)
Age (years)	50 (6)	49 (6)	48 (6)	47 (5)
Race (%)				
Mexican American	154 (27.8)	143 (25.9)	136 (24.6)	81 (14.7)
Other Hispanic	25 (4.5)	26 (4.7)	34 (6.2)	27 (4.9)
White	282 (51.0)	283 (51.2)	270 (48.9)	277 (50.2)
Black	81 (14.6)	83 (15.0)	91 (16.5)	155 (28.1)
Other	11 (2.0)	18 (3.3)	21 (3.8)	12 (2.2)
Education level (%)				
Less Than 9th Grade	87 (15.7)	77 (13.9)	63 (11.4)	48 (8.7)
9-11th Grade	86 (15.6)	80 (14.5)	115 (20.8)	82 (14.9)
High School Graduate	123 (22.2)	116 (21.0)	115 (20.8)	115 (20.8)
Some College or AA degree	151 (27.3)	161 (29.2)	119 (21.6)	164 (29.7)
College Graduate or above	106 (19.2)	118 (21.4)	140 (25.4)	143 (25.9)
Marital status (%)				
Lives alone	152 (28.4)	155 (29.1)	140 (26.9)	167 (32.9)
Lives with a partner	384 (71.6)	378 (70.9)	380 (73.1)	341 (67.1)
PIR	3.16 [1.40, 5.00]	3.16 [1.48, 5.00]	2.99 [1.36, 5.00]	3.23 [1.69, 5.00]
Height (cm)	169.0 (9.9)	168.6 (10.0)	168.2 (10.5)	169.1 (9.8)
BMI (kg/m^2)	29.69 (6.97)	29.28 (6.88)	29.03 (6.32)	28.75 (6.44)
Waist circumference (cm)	100.0 (15.9)	98.9 (15.4)	97.9 (15.0)	97.1 (15.1)
Systolic blood pressure (mmHg)	126 (17)	123 (17)	123 (18)	124 (17)
Diastolic blood pressure (mmHg)	77 (12)	76 (11)	76 (12)	77 (10)
Active smoking ((cigarettes/day)*years)	0 [0, 0]	0 [0, 0]	0 [0, 0]	0 [0, 0]
Passive smoking (cigarettes/day)	0 [0, 0]	0 [0, 0]	0 [0, 0]	0 [0, 0]
PA level (%)				
1	148 (26.8)	122 (22.1)	129 (23.4)	132 (24.0)
2	270 (48.8)	288 (52.1)	295 (53.4)	295 (53.5)
3	95 (17.2)	96 (17.4)	87 (15.8)	82 (14.9)
4	40 (7.2)	47 (8.5)	41 (7.4)	42 (7.6)
PA MET (MET*minutes*frequency)	40 [0, 408]	80 [0, 437]	60 [0, 481]	108 [0, 469]

Abbreviations: MET, metabolic equivalent of task; PA, physical activity; PIR, poverty to income ratio; Q, quartile.
[1]Continuous variables are expressed as mean (SD) or median [Q1, Q3]. Categorical variables are expressed as counts (%). In the left column, in parenthesis, the units of measure are reported; this does not apply to pure numbers.

Supplementary Table 1. Old. Demographic, examination and lifestyle characteristics of the Old group, stratified by telomere length quartiles.[1]

	Q1	Q2	Q3	Q4
n	566	566	566	565
Telomere length (T/S ratio)	0.67 (0.06)	0.82 (0.03)	0.95 (0.04)	1.19 (0.16)
NHANES cycle (%)				
1999-2000	325 (57.4)	252 (44.5)	249 (44.0)	277 (49.0)
2001-2002	241 (42.6)	314 (55.5)	317 (56.0)	288 (51.0)
Sex (%)				
Female	245 (43.3)	266 (47.0)	287 (50.7)	326 (57.7)
Male	321 (56.7)	300 (53.0)	279 (49.3)	239 (42.3)
Age (years)	72 (7)	71 (7)	70 (7)	68 (6)
Race (%)				
Mexican American	144 (25.4)	112 (19.8)	125 (22.1)	112 (19.8)
Other Hispanic	21 (3.7)	22 (3.9)	20 (3.5)	23 (4.1)
White	329 (58.1)	341 (60.2)	319 (56.4)	322 (57.0)
Black	64 (11.3)	75 (13.3)	87 (15.4)	99 (17.5)
Other	8 (1.4)	16 (2.8)	15 (2.7)	9 (1.6)
Education level (%)				
Less Than 9th Grade	158 (27.9)	140 (24.8)	129 (22.8)	122 (21.6)
9-11th Grade	102 (18.0)	101 (17.9)	98 (17.3)	89 (15.8)
High School Graduate	126 (22.3)	128 (22.7)	131 (23.2)	152 (26.9)
Some College or AA degree	108 (19.1)	103 (18.2)	111 (19.6)	113 (20.0)
College Graduate or above	72 (12.7)	93 (16.5)	96 (17.0)	89 (15.8)
Marital status (%)				
Lives alone	198 (35.8)	186 (34.0)	187 (34.6)	180 (34.4)
Lives with a partner	355 (64.2)	361 (66.0)	354 (65.4)	344 (65.6)
PIR	1.97 [1.19, 3.39]	2.22 [1.21, 3.83]	2.25 [1.24, 4.07]	2.58 [1.35, 4.42]
Height (cm)	165.9 (9.6)	166.0 (9.9)	165.4 (10.1)	165.3 (10.0)
BMI (kg/m²)	28.08 (5.12)	28.52 (5.43)	28.37 (5.40)	28.81 (5.69)
Waist circumference (cm)	100.5 (13.6)	101.0 (13.7)	100.0 (13.1)	99.9 (13.7)
Systolic blood pressure (mmHg)	141 (23)	138 (22)	138 (22)	141 (21)
Diastolic blood pressure (mmHg)	69 (17)	68 (16)	70 (16)	71 (15)
Active smoking ((cigarettes/day)*years)	0 [0, 0]	0 [0, 0]	0 [0, 0]	0 [0, 0]
Passive smoking (cigarettes/day)	0 [0, 0]	0 [0, 0]	0 [0, 0]	0 [0, 0]
PA level (%)				
1	172 (30.4)	149 (26.4)	147 (26.0)	144 (25.6)
2	327 (57.9)	336 (59.5)	312 (55.2)	334 (59.4)
3	56 (9.9)	60 (10.6)	87 (15.4)	73 (13.0)
4	10 (1.8)	20 (3.5)	19 (3.4)	11 (2.0)
PA MET (MET*minutes*frequency)	0 [0, 303]	0 [0, 295]	0 [0, 359]	0 [0, 397]

Abbreviations: MET, metabolic equivalent of task; PA, physical activity; PIR, poverty to income ratio; Q, quartile.
[1]Continuous variables are expressed as mean (SD) or median [Q1, Q3]. Categorical variables are expressed as counts (%). In the left column, in parenthesis, the units of measure are reported; this does not apply to pure numbers.

Supplementary Table 2. Young. Laboratory variables of the Young group, stratified by telomere length quartiles.[1]

	Q1	Q2	Q3	Q4
n	656	656	656	655
Telomere length (T/S ratio)	0.85 (0.08)	1.04 (0.04)	1.19 (0.05)	1.45 (0.15)
Total cholesterol (mg/dL)	197 (44)	197 (42)	194 (42)	192 (42)
HDL (mg/dL)	50 (15)	51 (15)	52 (15)	52 (15)
C-reactive protein (mg/dL)	0.24 [0.08, 0.55]	0.22 [0.08, 0.47]	0.18 [0.06, 0.47]	0.17 [0.06, 0.46]
gHb (%)	5.1 [4.9, 5.3]	5.1 [4.9, 5.3]	5.1 [5.0, 5.3]	5.1 [4.9, 5.3]
Leukocyte count (SI)	7.7 (2.3)	7.8 (2.3)	7.7 (2.3)	7.4 (2.3)
Lymphocytes (%)	29.1 (8.3)	28.8 (8.5)	29.3 (8.5)	29.7 (8.8)
Monocytes (%)	7.8 (2.0)	7.7 (2.1)	7.7 (2.3)	7.9 (2.4)
Neutrophils (%)	60.0 (9.8)	60.4 (10.0)	59.9 (9.9)	59.3 (10.1)
Eosinophils (%)	2.0 [1.3, 3.2]	2.0 [1.2, 3.1]	2.0 [1.2, 3.2]	2.0 [1.3, 3.3]
Basophils (%)	0.6 [0.4, 0.7]	0.6 [0.4, 0.8]	0.6 [0.4, 0.8]	0.6 [0.4, 0.8]
Erythrocyte count (SI)	4.69 (0.57)	4.67 (0.56)	4.69 (0.56)	4.70 (0.59)
Hb (g/dL)	14.2 (1.7)	14.1 (1.7)	14.2 (1.7)	14.1 (1.7)
Hematocrit (%)	41.8 (4.9)	41.6 (4.9)	41.7 (4.9)	41.7 (5.1)
Platelet count (SI)	273 (66)	271 (64)	270 (64)	271 (67)
Iron (µg/dL)	142 (91)	138 (116)	127 (80)	128 (102)
TIBC (µg/dL)	118 (33)	114 (33)	116 (36)	119 (38)
Transferrin saturation (%)	93.2 (14.3)	95.4 (35.5)	93.8 (28.8)	92.8 (23.9)
Ferritin (ng/mL)	62 [25, 133]	59 [21, 125]	53 [22, 124]	54 [24, 121]
Folate (ng/mL)	11.4 [8.4, 16.6]	11.3 [8.4, 16.0]	11.1 [8.3, 15.6]	11.4 [8.1, 16.5]
Cobalamin (pg/mL)	434 [331, 565]	441 [340, 564]	444 [334, 562]	437 [339, 590]
Homocysteine (µmol/L)	6.66 [5.23, 8.10]	6.58 [5.24, 7.90]	6.72 [5.28, 8.14]	6.53 [5.29, 8.15]
Methylmalonic acid (µmol/L)	0.11 [0.09, 0.15]	0.11 [0.09, 0.14]	0.11 [0.09, 0.15]	0.11 [0.09, 0.15]
Cotinine (ng/mL)	0.13 [0.04, 38.43]	0.12 [0.04, 21.00]	0.12 [0.04, 29.31]	0.18 [0.04, 65.66]
γ-tocopherol (µg/dL)	222.2 [158.1, 295.3]	225.9 [165.9, 300.2]	212.0 [150.3, 284.0]	215.0 [150.6, 282.6]
Retinyl palmitate (µg/dL)	1.70 [1.10, 2.80]	1.80 [1.10, 2.90]	1.80 [1.10, 2.80]	1.80 [1.10, 2.81]
Retinyl stearate (µg/dL)	0.35 [0.35, 0.35]	0.35 [0.35, 0.35]	0.35 [0.35, 0.35]	0.35 [0.35, 0.35]
Vitamin A (µg/dL)	53.2 (16.0)	52.2 (14.5)	51.9 (14.8)	52.6 (15.5)
Vitamin E (µg/dL)	1031.6 [819.1, 1275.6]	996.7 [853.9, 1255.8]	970.9 [806.8, 1219.3]	945.7 [796.3, 1166.8]
Triglycerides (mg/dL)	114 [78, 182]	111 [72, 162]	104 [72, 155]	99 [70, 167]
LDL (mg/dL)	119 (33)	114 (33)	116 (36)	119 (38)
Glucose (mg/dL)	92.2 [86.3, 98.4]	91.1 [84.9, 98.9]	89.8 [84.1, 96.2]	90.0 [85.6, 96.7]
C-peptide (nmol/L)	0.71 [0.51, 0.94]	0.66 [0.48, 0.90]	0.63 [0.46, 0.88]	0.63 [0.49, 0.82]
Insulin (µU/mL)	9.80 [6.94, 15.48]	10.10 [6.85, 14.35]	9.16 [6.77, 14.21]	9.36 [6.79, 13.54]

Abbreviations: gHb, glycated hemoglobin; Hb, hemoglobin; Q, quartile; TIBC, total iron binding capacity.
[1]The variables are all continuous, and they are expressed as mean (SD) or median [Q1, Q3]. In the left column, in parenthesis, the units of measure are reported.

Supplementary Table 2. Middle. Laboratory variables of the Middle group, stratified by telomere length quartiles.[1]

	Q1	Q2	Q3	Q4
n	553	553	552	552
Telomere length (T/S ratio)	0.76 (0.08)	0.93 (0.04)	1.07 (0.04)	1.34 (0.15)
Total cholesterol (mg/dL)	211 (45)	209 (38)	211 (42)	210 (41)
HDL (mg/dL)	51 (15)	50 (15)	51 (15)	52 (16)
C-reactive protein (mg/dL)	0.26 [0.12, 0.60]	0.23 [0.10, 0.50]	0.25 [0.10, 0.54]	0.20 [0.08, 0.47]
gHb (%)	5.4 [5.2, 5.7]	5.4 [5.2, 5.6]	5.4 [5.1, 5.6]	5.4 [5.1, 5.7]
Leukocyte count (SI)	7.2 (2.4)	7.1 (2.1)	7.2 (2.0)	6.9 (2.0)
Lymphocytes (%)	31.2 (8.6)	30.5 (7.9)	30.8 (7.9)	31.7 (8.5)
Monocytes (%)	8.2 (2.1)	8.0 (2.1)	7.9 (2.1)	8.0 (2.1)
Neutrophils (%)	57.2 (9.4)	58.1 (8.7)	57.8 (8.7)	56.9 (9.4)
Eosinophils (%)	2.3 [1.6, 3.4]	2.3 [1.5, 3.5]	2.3 [1.6, 3.5]	2.3 [1.5, 3.5]
Basophils (%)	0.6 [0.4, 0.9]	0.6 [0.4, 0.9]	0.6 [0.4, 0.9]	0.6 [0.4, 0.9]
Erythrocyte count (SI)	4.78 (0.49)	4.75 (0.45)	4.71 (0.48)	4.71 (0.52)
Hb (g/dL)	14.6 (1.6)	14.5 (1.5)	14.4 (1.5)	14.2 (1.5)
Hematocrit (%)	43.1 (4.5)	42.6 (4.2)	42.4 (4.2)	42.0 (4.4)
Platelet count (SI)	268 (70)	276 (69)	271 (67)	274 (67)
Iron (µg/dL)	157 (115)	162 (117)	192 (283)	148 (144)
TIBC (µg/dL)	129 (34)	130 (34)	127 (34)	128 (35)
Transferrin saturation (%)	106.4 (33.8)	107.5 (32.7)	107.0 (39.6)	99.9 (20.6)
Ferritin (ng/mL)	107 [46, 206]	93 [44, 196]	94 [40, 174]	94 [36, 205]
Folate (ng/mL)	12.1 [8.7, 16.6]	12.0 [8.7, 16.4]	12.2 [8.9, 16.6]	12.7 [9.2, 17.7]
Cobalamin (pg/mL)	463 [362, 608]	459 [350, 617]	460 [352, 614]	484 [373, 627]
Homocysteine (µmol/L)	7.82 [6.64, 9.69]	7.96 [6.70, 9.48]	7.78 [6.28, 9.56]	7.77 [6.47, 9.65]
Methylmalonic acid (µmol/L)	0.12 [0.10, 0.16]	0.12 [0.10, 0.16]	0.12 [0.10, 0.16]	0.12 [0.10, 0.16]
Cotinine (ng/mL)	0.10 [0.04, 71.07]	0.10 [0.04, 99.68]	0.10 [0.04, 76.40]	0.11 [0.04, 43.83]
γ-tocopherol (µg/dL)	245.0 [164.0, 348.0]	230.0 [163.0, 322.0]	234.0 [152.0, 312.8]	219.5 [137.4, 316.0]
Retinyl palmitate (µg/dL)	1.70 [1.00, 2.80]	1.80 [1.20, 2.77]	1.90 [1.20, 3.00]	1.90 [1.10, 3.10]
Retinyl stearate (µg/dL)	0.35 [0.35, 0.35]	0.35 [0.35, 0.35]	0.35 [0.35, 0.35]	0.35 [0.35, 0.35]
Vitamin A (µg/dL)	59.2 (17.77)	60.0 (18.00)	60.4 (17.27)	60.1 (17.5)
Vitamin E (µg/dL)	1203.3 [951.6, 1549.4]	1230.5 [1009.4, 1496.1]	1207.1 [1000.0, 1553.7]	1201.6 [960.6, 1578.6]
Triglycerides (mg/dL)	132 [87, 187]	129 [91, 204]	129 [89, 193]	114 [77, 169]
LDL (mg/dL)	129 (34)	130 (34)	127 (34)	128 (35)
Glucose (mg/dL)	98.4 [91.8, 106.0]	98.9 [92.6, 108.1]	98.6 [90.7, 107.4]	95.6 [89.2, 103.9]
C-peptide (nmol/L)	0.78 [0.59, 1.09]	0.76 [0.57, 1.08]	0.74 [0.55, 1.01]	0.65 [0.50, 1.02]
Insulin (µU/mL)	11.55 [7.63, 17.04]	10.80 [7.43, 16.13]	10.19 [7.26, 15.44]	9.41 [6.78, 14.77]

Abbreviations: gHb, glycated hemoglobin; Hb, hemoglobin; Q, quartile; TIBC, total iron binding capacity.
[1]The variables are all continuous, and they are expressed as mean (SD) or median [Q1, Q3]. In the left column, in parenthesis, the units of measure are reported.

Supplementary Table 2. Old. Laboratory variables of the Old group, stratified by telomere length quartiles.[1]

	Q1	Q2	Q3	Q4
n	566	566	566	565
Telomere length (T/S ratio)	0.67 (0.06)	0.82 (0.03)	0.95 (0.04)	1.19 (0.16)
Total cholesterol (mg/dL)	209 (44)	209 (39)	213 (39)	212 (40)
HDL (mg/dL)	51 (16)	53 (16)	53 (16)	54 (17)
C-reactive protein (mg/dL)	0.32 [0.14, 0.62]	0.28 [0.14, 0.64]	0.28 [0.14, 0.55]	0.28 [0.12, 0.59]
gHb (%)	5.6 [5.3, 6.0]	5.6 [5.3, 6.1]	5.6 [5.3, 6.0]	5.6 [5.3, 6.0]
Leukocyte count (SI)	6.9 [5.9, 8.3]	6.9 [5.9, 8.2]	6.9 [5.7, 8.0]	6.6 [5.5, 8.0]
Lymphocytes (%)	28.9 (9.0)	28.2 (8.7)	29.0 (8.0)	29.3 (9.0)
Monocytes (%)	8.7 (2.2)	8.5 (2.1)	8.3 (2.2)	8.5 (2.3)
Neutrophils (%)	58.7 (9.7)	59.7 (9.2)	59.0 (8.7)	58.7 (9.8)
Eosinophils (%)	2.7 [1.7, 3.8]	2.6 [1.7, 3.7]	2.3 [1.7, 3.6]	2.4 [1.7, 3.6]
Basophils (%)	0.6 [0.4, 0.8]	0.6 [0.4, 0.8]	0.6 [0.4, 0.0]	0.6 [0.4, 0.9]
Erythrocyte count (SI)	4.60 (0.52)	4.60 (0.47)	4.67 (0.47)	4.61 (0.47)
Hb (g/dL)	14.2 (1.4)	14.2 (1.4)	14.3 (1.4)	14.1 (1.3)
Hematocrit (%)	42.1 (4.3)	42.1 (4.1)	42.2 (4.1)	41.8 (4.0)
Platelet count (SI)	256 (84)	254 (70)	259 (73)	258 (61)
Iron (µg/dL)	172 (118)	158 (86)	165 (95)	151 (80)
TIBC (µg/dL)	124 (36)	125 (31)	133 (37)	127 (35)
Transferrin saturation (%)	116.4 (42.6)	116.3 (43.0)	113.4 (46.2)	112.9 (34.4)
Ferritin (ng/mL)	122 [63, 221]	119 [63, 224]	100 [55, 183]	100 [55, 173]
Folate (ng/mL)	15.6 [10.7, 23.4]	15.8 [11.1, 22.6]	15.0 [10.6, 20.9]	16.8 [11.7, 22.7]
Cobalamin (pg/mL)	469 [342, 663]	465 [345, 634]	487 [359, 632]	481 [362, 659]
Homocysteine (µmol/L)	9.72 [8.08, 11.94]	9.57 [7.82, 11.79]	9.37 [7.55, 11.41]	8.75 [7.27, 11.12]
Methylmalonic acid (µmol/L)	0.16 [0.12, 0.22]	0.16 [0.12, 0.21]	0.14 [0.12, 0.20]	0.14 [0.11, 0.19]
Cotinine (ng/mL)	0.04 [0.04, 0.44]	0.04 [0.03, 0.26]	0.05 [0.04, 0.46]	0.04 [0.04, 0.19]
γ-tocopherol (µg/dL)	211.0 [124.9, 322.9]	200.7 [120.1, 306.0]	189.0 [105.2, 303.4]	194.0 [106.7, 298.0]
Retinyl palmitate (µg/dL)	1.50 [0.90, 2.70]	1.80 [1.10, 2.90]	1.80 [1.00, 2.80]	1.80 [1.00, 3.10]
Retinyl stearate (µg/dL)	0.35 [0.35, 0.35]	0.35 [0.35, 0.35]	0.35 [0.35, 0.35]	0.35 [0.35, 0.35]
Vitamin A (µg/dL)	64.6 (21.5)	66.1 (20.7)	63.8 (17.6)	64.4 (17.9)
Vitamin E (µg/dL)	1378.3 [1084.9, 1903.2]	1382.9 [1101.0, 1902.9]	1417.0 [1080.0, 1980.8]	1428.5 [1098.3, 1909.4]
Triglycerides (mg/dL)	145 [103, 201]	138 [102, 191]	135 [101, 207]	132 [95, 190]
LDL (mg/dL)	123 (36)	125 (31)	132 (37)	127 (35)
Glucose (mg/dL)	102.8 [94.3, 115.7]	103.8 [94.9, 117.7]	103.0 [94.6, 114.2]	103.2 [94.5, 116.3]
C-peptide (nmol/L)	0.88 [0.63, 1.19]	0.87 [0.62, 1.20]	0.82 [0.62, 1.12]	0.86 [0.60, 1.16]
Insulin (µU/mL)	11.37 [7.85, 17.00]	11.43 [7.61, 18.18]	10.77 [7.56, 18.18]	11.34 [7.40, 17.59]

Abbreviations: gHb, glycated hemoglobin; Hb, hemoglobin; Q, quartile; TIBC, total iron binding capacity.
[1]The variables are all continuous, and they are expressed as mean (SD) or median [Q1, Q3]. In the left column, in parenthesis, the units of measure are reported.
