# Supplementary material for: Diet, lifestyle and telomere length: using Copula Graphical Models on NHANES data
Source: Aging (Albany NY). 2025 Jan 29;17(2):329–56. doi: 10.18632/aging.206194 (PMC11892917; doi:10.18632/aging.206194)
Supplement: Supplementary Table 3 [file aging-17-206194-s002.docx]

**Supplementary Table 3 – Young**Values of the partial correlations between telomere length and the variables with a certainty ≥ 0.95 in the main analysis of the Young group.^1^

|  | Main analysis | Males | Females | Mexicans | Whites | Blacks | PIR < Mdn | PIR ≥ Mdn | BMI < 25 | 25 ≤ BMI  < 30 | BMI ≥ 30 | NHANES 1999-2000 | NHANES 2001-2002 | All study population |
| --- | --- | --- | --- | --- | --- | --- | --- | --- | --- | --- | --- | --- | --- | --- |
| *n* | 2623 | 1126 | 1497 | 637 | 1343 | 421 | 1213 | 1219 | 987 | 857 | 750 | 1193 | 1430 | 7096 |
| NHANES cycle | 0.085 | 0.066 | 0.198 | 0.103 | 0.160 | 0.049 | 0.147 | 0.110 | 0.100 | 0.117 | 0.105 | - | - | 0.113 |
| Age | -0.107 | -0.108 | -0.097 | -0.082 | -0.083 | -0.094 | -0.116 | -0.076 | -0.090 | -0.054 | -0.100 | -0.089 | -0.117 | -0.320 |
| PA level | 0.032 | 0 | 0.075 | 0.035 | 0.020 | 0 | 0.056 | 0.008 | 0.005 | 0 | 0.019 | 0.050 | 0 | 0.026 |
| C-reactive protein | -0.034 | -0.025 | 0 | 0 | -0.020 | -0.003 | -0.021 | 0 | 0 | -0.023 | 0 | -0.010 | 0 | -0.064 |
| Basophils | 0.057 | 0.025 | 0.032 | 0 | 0.047 | 0 | 0.033 | 0.025 | 0.005 | 0 | 0.074 | 0 | 0.054 | 0.062 |
| Erythrocyte count | 0.024 | 0.043 | 0 | 0 | 0 | 0 | 0 | 0 | 0 | 0.011 | 0 | 0.007 | 0 | 0.052 |
| Hb | -0.015 | -0.003 | 0 | -0.012 | 0 | 0 | -0.013 | 0 | 0 | 0 | -0.012 | -0.009 | 0 | -0.040 |
| TIBC | -0.041 | -0.027 | -0.045 | 0 | -0.069 | 0 | -0.031 | -0.015 | -0.036 | 0 | 0 | 0 | -0.069 | -0.042 |
| γ-tocopherol | -0.040 | 0 | -0.058 | -0.077 | -0.002 | 0 | -0.042 | -0.009 | -0.008 | -0.032 | 0 | -0.065 | 0 | -0.070 |
| Retinyl palmitate | -0.031 | 0 | -0.011 | 0 | 0 | 0 | -0.020 | 0 | -0.039 | 0.008 | 0 | -0.005 | 0 | -0.028 |
| Retinyl stearate | 0.040 | 0.106 | 0.188 | 0.074 | 0.112 | 0.111 | 0.134 | 0.130 | 0.134 | 0.114 | 0.101 | 0.377 | -0.030 | 0.068 |
| Insulin^2^ | 0.015 | 0.025 | 0.033 | 0.009 | 0.042 | 0.021 | 0.030 | 0.001 | 0.046 | 0.046 | 0.002 | 0.037 | 0.024 | 0.038 |
| Dietary fibre^3^ | -0.032 | -0.037 | -0.005 | 0 | -0.019 | 0 | -0.041 | 0 | 0 | -0.024 | 0 | -0.034 | 0 | -0.047 |
| Sodium^3^ | 0.027 | 0 | 0.013 | 0 | 0.011 | 0.002 | 0.002 | 0 | 0 | 0 | 0 | 0.008 | 0 | 0.018 |
| Caffeine^3^ | -0.045 | -0.003 | -0.072 | -0.008 | -0.026 | -0.039 | -0.066 | -0.008 | -0.028 | 0 | -0.056 | -0.046 | -0.041 | -0.049 |
| PFA 22:5^3^ | 0.012 | 0.022 | 0.002 | 0.030 | 0.003 | 0 | 0.002 | 0 | 0.030 | 0 | 0 | 0 | 0.028 | 0.012 |

Abbreviations: Hb, hemoglobin; PA, physical activity; PFA 22:5, Docosapentaenoic acid; TIBC, total iron binding capacity.
^1^ The name of each row indicates the variables that, in the main analysis, are related with telomere length with a certainty ≥ 0.95. In the first column are reported the values of the partial correlations between telomere length and the variables in the main analysis; in the other columns, the same is done for each sensitivity analysis.
All the values present in the table are taken from the optimal partial correlation matrix that was generated with the function “nutriNetwork”, and then selected with the function “selectnet”.
^2^ “Insulin” is colored in red to indicate that it is a variable measured in half of the sample; the participants belonging to this half were requested to come to the examination after at least 8.5 hours of fasting.
^3^ The green color indicates the dietary variables, in order not to confuse them with the other variables.

**Supplementary Table 3 – Middle**Values of the partial correlations between telomere length and the variables with a certainty ≥ 0.95 in the main analysis of the Middle group.^1^

|  | Main analysis | Males | Females | Mexicans | Whites | Blacks | PIR < Mdn | PIR ≥ Mdn | BMI < 25 | 25 ≤ BMI  < 30 | BMI ≥ 30 | NHANES 1999-2000 | NHANES 2001-2002 | All study population |
| --- | --- | --- | --- | --- | --- | --- | --- | --- | --- | --- | --- | --- | --- | --- |
| *n* | 2210 | 1133 | 1077 | 537 | 1110 | 365 | 1019 | 1020 | 581 | 807 | 793 | 952 | 1258 | 7096 |
| NHANES cycle | 0.106 | 0.118 | 0.156 | 0.086 | 0.153 | 0.069 | 0.055 | 0.201 | 0.113 | 0.087 | 0.131 | - | - | 0.113 |
| Age | -0.136 | -0.128 | -0.113 | -0.103 | -0.076 | -0.091 | -0.097 | -0.135 | -0.136 | -0.091 | -0.091 | -0.111 | -0.157 | -0.320 |
| Education level | 0.051 | 0.052 | 0.016 | 0.028 | 0.039 | 0.027 | 0.070 | 0.059 | 0.016 | 0.040 | 0.007 | 0.022 | 0.046 | 0.043 |
| C-reactive protein | -0.055 | -0.053 | -0.006 | 0 | -0.064 | -0.005 | -0.047 | 0 | -0.005 | -0.080 | 0 | -0.041 | -0.012 | -0.064 |
| Basophils | 0.038 | 0.024 | 0 | 0 | 0.064 | 0 | 0.011 | 0 | 0 | 0 | 0.004 | 0.065 | 0 | 0.062 |
| Hb | -0.034 | -0.030 | -0.012 | -0.021 | -0.022 | -0.007 | -0.040 | -0.028 | -0.005 | -0.044 | -0.022 | -0.060 | -0.010 | -0.040 |
| TIBC | -0.060 | -0.071 | -0.050 | -0.008 | -0.025 | -0.073 | -0.036 | -0.052 | 0 | -0.041 | -0.022 | -0.047 | -0.060 | -0.042 |
| Folate | 0.032 | 0 | 0.001 | 0 | 0 | 0 | 0 | 0 | 0 | 0 | 0 | 0 | 0.024 | 0.022 |
| γ-tocopherol | -0.070 | -0.062 | -0.097 | -0.048 | -0.068 | -0.056 | -0.058 | -0.063 | -0.037 | -0.096 | -0.008 | -0.133 | -0.040 | -0.070 |
| Retinyl stearate | 0.040 | 0.093 | 0.156 | 0.058 | 0.176 | 0 | 0.068 | 0.142 | 0.113 | 0.110 | 0.037 | 0.343 | -0.009 | 0.068 |
| Vitamin A | 0.030 | 0.011 | 0.017 | 0 | 0.020 | 0 | 0 | 0.041 | 0 | 0 | 0.032 | 0.012 | 0.003 | 0.026 |
| Caffeine^2^ | -0.038 | -0.006 | -0.039 | -0.019 | -0.022 | 0 | 0 | -0.050 | 0 | -0.027 | -0.003 | 0 | -0.045 | -0.049 |

Abbreviations: Hb, hemoglobin; TIBC, total iron binding capacity.
^1^ The name of each row indicates the variables that, in the main analysis, are related with telomere length with a certainty ≥ 0.95. In the first column are reported the values of the partial correlations between telomere length and the variables in the main analysis; in the other columns, the same is done for each sensitivity analysis.
All the values present in the table are taken from the optimal partial correlation matrix that was generated with the function “nutriNetwork”, and then selected with the function “selectnet”.
^2^ The green color indicates the only dietary variable, in order not to confuse it with the other variables.

**Supplementary Table 3 – Old**Values of the partial correlations between telomere length and the variables with a certainty ≥ 0.95 in the main analysis of the Old group.^1^

|  | Main analysis | Males | Females | Mexicans | Whites | Blacks | PIR < Mdn | PIR ≥ Mdn | BMI < 25 | 25 ≤ BMI  < 30 | BMI ≥ 30 | NHANES 1999-2000 | NHANES 2001-2002 | All study population |
| --- | --- | --- | --- | --- | --- | --- | --- | --- | --- | --- | --- | --- | --- | --- |
| *n* | 2263 | 1139 | 1124 | 543 | 1151 | 394 | 998 | 1013 | 590 | 865 | 725 | 1103 | 1160 | 7096 |
| NHANES cycle | 0.059 | 0.087 | 0.094 | 0.077 | 0.105 | 0 | 0.150 | 0.012 | 0.046 | 0.058 | 0.062 | - | - | 0.113 |
| Sex | 0.082 | - | - | 0.057 | 0.065 | 0 | 0.055 | 0.057 | 0 | 0.022 | 0.064 | 0.041 | 0.071 | 0.062 |
| Age | -0.167 | -0.157 | -0.141 | -0.119 | -0.145 | -0.070 | -0.166 | -0.152 | -0.096 | -0.148 | -0.130 | -0.165 | -0.159 | -0.320 |
| PIR | 0.058 | 0.089 | 0.087 | 0.052 | 0.068 | 0.051 | 0 | 0.026 | 0.100 | 0.103 | 0.028 | 0.128 | 0.041 | 0.029 |
| Leukocyte count | -0.073 | -0.102 | -0.095 | -0.077 | -0.130 | 0 | -0.059 | -0.102 | -0.070 | -0.102 | 0 | -0.127 | -0.082 | -0.045 |
| Basophils | 0.034 | 0 | 0.023 | 0 | 0 | 0.024 | 0 | 0 | 0 | 0 | 0 | 0.029 | 0 | 0.062 |
| Erythrocyte count | 0.046 | 0.053 | 0.012 | 0 | 0.031 | 0 | 0 | 0.075 | 0 | 0.010 | 0 | 0.025 | 0.046 | 0.052 |
| TIBC | -0.036 | 0 | -0.007 | 0 | -0.022 | 0 | 0 | -0.040 | 0 | 0 | 0 | 0 | -0.030 | -0.042 |
| Ferritin | -0.057 | -0.064 | 0 | -0.037 | -0.020 | 0 | 0 | -0.075 | -0.022 | -0.013 | 0 | -0.001 | -0.083 | -0.023 |
| γ-tocopherol | -0.045 | -0.018 | 0 | 0 | -0.012 | 0 | 0 | 0 | 0 | 0 | -0.001 | 0 | -0.064 | -0.070 |
| Retinyl stearate | 0.048 | 0.177 | 0.102 | 0.110 | 0.108 | 0.095 | 0.112 | 0.109 | 0.111 | 0.097 | 0.107 | 0.380 | -0.040 | 0.068 |
| LDL | 0.033 | 0.262 | 0.190 | 0.181 | 0.235 | 0.172 | 0.264 | 0.158 | 0.169 | 0.191 | 0.218 | 0.273 | 0.226 | 0.040 |
| MFA 20:1^2^ | 0.032 | 0.015 | 0.010 | 0.005 | 0 | 0 | 0 | 0.011 | 0 | 0 | 0.015 | 0.035 | 0.006 | 0.008 |

Abbreviations: MFA 20:1, Eicosenoic acid; PIR, poverty to income ratio; TIBC, total iron binding capacity.
^1^ The name of each row indicates the variables that, in the main analysis, are related with telomere length with a certainty ≥ 0.95. In the first column are reported the values of the partial correlations between telomere length and the variables in the main analysis; in the other columns, the same is done for each sensitivity analysis.
All the values present in the table are taken from the optimal partial correlation matrix that was generated with the function “nutriNetwork”, and then selected with the function “selectnet”.
^2^ The green color indicates the only dietary variable, in order not to confuse it with the other variables.
