# Supplementary material for: Diet, lifestyle and telomere length: using Copula Graphical Models on NHANES data
Source: Aging (Albany NY). 2025 Jan 29;17(2):329–56. doi: 10.18632/aging.206194 (PMC11892917; doi:10.18632/aging.206194)
Supplement: Supplementary Tables 4-6 [file aging-17-206194-s003.pdf]

Supplementary Table 4. Characteristics of the whole population after stratification in quartiles of caffeine intake.[1]

	Q1	Q2	Q3	Q4
n	1774	1774	1774	1774
Telomere length (T/S ratio)	1.05 (0.25)	1.04 (0.25)	1.01 (0.24)	1.01 (0.23)
NHANES cycle (%)				
1999-2000	784 (44.2)	828 (46.7)	739 (41.7)	897 (50.6)
2001-2002	990 (55.8)	946 (53.3)	1035 (58.3)	877 (49.4)
Sex (%)				
Female	726 (40.9)	791 (44.6)	857 (48.3)	1024 (57.7)
Male	1048 (59.1)	983 (55.4)	917 (51.7)	750 (42.3)
Age (years)	47 (19)	47 (19)	49 (18)	50 (15)
BMI (kg/m^2)	28.30 (6.36)	28.45 (6.08)	28.51 (6.20)	28.50 (6.17)
Waist circumference (cm)	96.1 (15.2)	96.8 (14.7)	97.7 (15.4)	98.2 (15.4)
Active smoking ((cigarettes/day)*years)	0 [0, 0]	0 [0, 0]	0 [0, 0]	0 [0, 14]
Passive smoking (cigarettes/day)	0 [0, 0]	0 [0, 0]	0 [0, 0]	0 [0, 3]
PA level (%)				
1	432 (24.4)	403 (22.7)	444 (25.1)	428 (24.2)
2	959 (54.1)	980 (55.3)	932 (52.6)	895 (50.5)
3	286 (16.1)	288 (16.3)	272 (15.3)	297 (16.8)
4	96 (5.4)	101 (5.7)	124 (7.0)	152 (8.6)
PA MET (MET*minutes*frequency)	36 [0, 423]	52 [0, 410]	54 [0, 433]	96 [0, 512]
Total cholesterol (mg/dL)	202 (42)	204 (43)	205 (41)	207 (43)
HDL (mg/dL)	53 (16)	51 (15)	51 (16)	51 (16)
C-reactive protein (mg/dL)	0.25 [0.09, 0.57]	0.25 [0.09, 0.57]	0.24 [0.09, 0.54]	0.22 [0.10, 0.47]
gHb (%)	5.3 [5.1, 5.6]	5.3 [5.1, 5.6]	5.4 [5.1, 5.7]	5.3 [5.1, 5.6]
Cotinine (ng/mL)	0.06 [0.04, 0.60]	0.06 [0.04, 0.77]	0.09 [0.04, 33.45]	0.19 [0.04, 140.00]
γ-tocopherol (μg/dL)	207.0 [134.5, 288.0]	216.0 [145.1, 300.0]	224.0 [153.0, 310.9]	229.0 [139.6, 320.7]
Vitamin A (μg/dL)	56.9 (18.2)	57.5 (18.9)	59.2 (18.1)	61.1 (17.2)
Vitamin E (μg/dL)	1165.0 [916.3, 1534.2]	1157.9 [918.0, 1533.1]	1153.1 [918.3, 1525.3]	1183.1 [942.8, 1569.1]
Dietary fiber (g)[2]	14.31 [9.35, 21.30]	13.96 [9.20, 20.96]	13.61 [8.89, 20.30]	14.52 [9.40, 21.04]
Energy (kcal)[2]	1812.47 [1341.40, 2389.37]	1929.65 [1431.36, 2554.75]	1987.08 [1473.37, 2670.25]	2188.35 [1617.25, 2907.63]
Caffeine (mg)[2]	0.00 [0.00, 3.00]	59.20 [37.82, 78.00]	159.48 [128.11, 191.63]	365.83 [289.17, 520.30]

Abbreviations: gHb, glycated hemoglobin; MET, metabolic equivalent of task; PA, physical activity; Q, quartile.
[1]Continuous variables are expressed as mean (SD) or median [Q1, Q3]. Categorical variables are expressed as counts (%). In the left column, in parenthesis, the units of measure are reported.
Only some of the demographic, examination, lifestyle, laboratory, and dietary variables are included.
[2]The green color indicates the dietary variables, in order not to confuse them with the other variables.

Supplementary Table 5. Characteristics of the whole population after stratification in quartiles of consumption of dietary fibres.[1]

	Q1	Q2	Q3	Q4
n	1774	1774	1774	1774
Telomere length (T/S ratio)	1.04 (0.25)	1.03 (0.24)	1.02 (0.24)	1.02 (0.24)
NHANES cycle (%)				
1999-2000	861 (48.5)	795 (44.8)	805 (45.4)	787 (44.4)
2001-2002	913 (51.5)	979 (55.2)	969 (54.6)	987 (55.6)
Sex (%)				
Female	681 (38.4)	754 (42.5)	863 (48.6)	1100 (62.0)
Male	1093 (61.6)	1020 (57.5)	911 (51.4)	674 (38.0)
Age (years)	47 (18)	49 (18)	49 (18)	49 (18)
BMI (kg/m²)	28.91 (6.70)	28.65 (6.34)	28.36 (6.03)	27.84 (5.64)
Waist circumference (cm)	97.4 (15.8)	97.0 (15.3)	97.4 (15.1)	97.0 (14.5)
Active smoking ((cigarettes/day)*years)	0 [0, 6]	0 [0, 0]	0 [0, 0]	0 [0, 0]
Passive smoking (cigarettes/day)	0 [0, 4]	0 [0, 0]	0 [0, 0]	0 [0, 0]
PA level (%)				
1	485 (27.4)	453 (25.6)	403 (22.7)	366 (20.7)
2	923 (52.1)	933 (52.7)	961 (54.2)	949 (53.6)
3	250 (14.1)	288 (16.3)	294 (16.6)	311 (17.6)
4	115 (6.5)	98 (5.5)	114 (6.4)	146 (8.2)
PA MET (MET*minutes*frequency)	0<br>[0, 315]	48<br>[0, 408]	96<br>[0, 472]	112<br>[0, 591]
Total cholesterol (mg/dL)	204 (41)	205 (43)	205 (44)	204 (41)
HDL (mg/dL)	52 (16)	52 (16)	52 (15)	51 (15)
C-reactive protein (mg/dL)	0.28<br>[0.11, 0.64]	0.25<br>[0.10, 0.56]	0.24<br>[0.10, 0.53]	0.19<br>[0.08, 0.41]
gHb (%)	5.3<br>[5.1, 5.6]	5.3<br>[5.1, 5.7]	5.3<br>[5.1, 5.6]	5.3<br>[5.1, 5.6]
Cotinine (ng/mL)	0.32<br>[0.04, 130.00]	0.09<br>[0.04, 27.19]	0.06<br>[0.04, 1.27]	0.04<br>[0.04, 0.40]
γ-tocopherol (µg/dL)	240.0<br>[167.8, 328.8]	226.0<br>[148.2, 314.0]	212.2<br>[137.0, 294.9]	193.2<br>[121.4, 285.0]
Vitamin A (µg/dL)	56.4 (18.7)	58.4 (18.2)	59.6 (18.0)	60.4 (17.6)
Vitamin E (µg/dL)	1069.4<br>[875.6, 1378.3]	1167.7<br>[922.2, 1550.1]	1201.9<br>[944.9, 1611.6]	1240.2<br>[969.3, 1632.1]
Dietary fiber (g)[2]	6.50<br>[4.80, 7.91]	11.60<br>[10.34, 12.85]	17.00<br>[15.50, 18.70]	27.20<br>[23.66, 33.60]
Energy (kcal)[2]	1436.50<br>[1097.25, 1868.56]	1825.55<br>[1424.17, 2404.00]	2141.00<br>[1657.22, 2698.24]	2617.59<br>[2040.50, 3354.81]
Caffeine (mg)[2]	106.05<br>[14.00, 223.80]	103.00<br>[15.48, 227.57]	105.28<br>[11.88, 251.00]	99.29<br>[9.07, 236.75]

Abbreviations: gHb, glycated hemoglobin; MET, metabolic equivalent of task; PA, physical activity; Q, quartile.
[1]Continuous variables are expressed as mean (SD) or median [Q1, Q3]. Categorical variables are expressed as counts (%). In the left column, in parenthesis, the units of measure are reported.
Only some of the demographic, examination, lifestyle, laboratory, and dietary variables are included.
[2] The green color indicates the dietary variables, in order not to confuse them with the other variables.

Supplementary Table 6. Characteristics of the whole population after stratification in quartiles of serum γ-tocopherol levels.[1]

	Q1	Q2	Q3	Q4
n	1660	1660	1660	1659
Telomere length (T/S ratio)	1.03 (0.25)	1.05 (0.25)	1.04 (0.24)	1.00 (0.23)
NHANES cycle (%)				
1999-2000	683 (41.1)	700 (42.2)	701 (42.2)	733 (44.2)
2001-2002	977 (58.9)	960 (57.8)	959 (57.8)	926 (55.8)
Sex (%)				
Female	790 (47.6)	793 (47.8)	793 (47.8)	806 (48.6)
Male	870 (52.4)	867 (52.2)	867 (52.2)	853 (51.4)
Age (years)	52 (19)	46 (18)	45 (17)	50 (17)
BMI (kg/m²)	26.59 (5.20)	27.46 (5.57)	28.88 (6.48)	30.90 (6.79)
Waist circumference (cm)	92.9 (14.3)	94.6 (14.3)	97.8 (15.1)	103.5 (15.3)
Active smoking ((cigarettes/day)*years)	0 [0, 0]	0 [0, 0]	0 [0, 0]	0 [0, 0]
Passive smoking (cigarettes/day)	0 [0, 0]	0 [0, 0]	0 [0, 0]	0 [0, 0]
PA level (%)				
1	374 (22.6)	380 (22.9)	390 (23.5)	457 (27.6)
2	917 (55.4)	887 (53.5)	863 (52.0)	842 (50.8)
3	286 (17.3)	271 (16.3)	291 (17.5)	229 (13.8)
4	79 (4.8)	120 (7.2)	116 (7.0)	130 (7.8)
PA MET (MET*minutes*frequency)	158 [0, 630]	63 [0, 441]	60 [0, 408]	0 [0, 315]
Total cholesterol (mg/dL)	194 (40)	196 (39)	204 (38)	223 (44)
HDL (mg/dL)	54 (16)	52 (15)	51 (15)	49 (16)
C-reactive protein (mg/dL)	0.19 [0.07, 0.43]	0.22 [0.09, 0.50]	0.24 [0.09, 0.52]	0.32 [0.16, 0.70]
gHb (%)	5.3 [5.1, 5.5]	5.3 [5.1, 5.6]	5.3 [5.1, 5.6]	5.5 [5.2, 5.9]
Cotinine (ng/mL)	0.04 [0.03, 0.40]	0.08 [0.04, 34.09]	0.14 [0.04, 37.68]	0.15 [0.04, 51.48]
γ-tocopherol (µg/dL)	96.7 [69.0, 121.0]	182.0 [163.0, 199.4]	259.0 [238.0, 281.8]	378.0 [337.0, 456.0]
Vitamin A (µg/dL)	62.2 (17.7)	57.9 (17.8)	57.0 (18.7)	57.9 (18.7)
Vitamin E (µg/dL)	1500.1 [1031.7, 2160.7]	1088.2 [868.4, 1415.0]	1056.5 [887.0, 1312.9]	1162.1 [953.6, 1451.6]
Dietary fiber (g)[2]	15.73 [10.70, 23.40]	14.33 [9.20, 21.30]	13.40 [8.72, 19.41]	12.65 [8.23, 18.88]
Energy (kcal)[2]	1911.00 [1443.50, 2512.75]	1990.80 [1462.70, 2645.50]	2038.34 [1506.42, 2767.90]	1965.00 [1445.50, 2669.01]
Caffeine (mg)[2]	91.00 [7.00, 250.08]	93.00 [9.28, 208.49]	100.00 [10.00, 223.40]	124.00 [36.90, 261.88]

Abbreviations: gHb, glycated hemoglobin; MET, metabolic equivalent of task; PA, physical activity; Q, quartile.
[1]Continuous variables are expressed as mean (SD) or median [Q1, Q3]. Categorical variables are expressed as counts (%). In the left column, in parenthesis, the units of measure are reported.
Only some of the demographic, examination, lifestyle, laboratory, and dietary variables are included.
[2]The green color indicates the dietary variables, in order not to confuse them with the other variables.
